# A New Approach for Investigating Iron Mineral Transformations in Soils and Sediments Using ^57^Fe-Labeled Minerals and ^57^Fe Mössbauer Spectroscopy

**DOI:** 10.1021/acs.est.3c00434

**Published:** 2023-06-26

**Authors:** Luiza Notini, Katrin Schulz, L. Joëlle Kubeneck, Andrew R. C. Grigg, Katherine A. Rothwell, Giulia Fantappiè, Laurel K. ThomasArrigo, Ruben Kretzschmar

**Affiliations:** Soil Chemistry Group, Institute of Biogeochemistry and Pollutant Dynamics, Department of Environmental Systems Science, ETH Zurich, CHN, Universitätstrasse 16, Zurich CH-8092, Switzerland

**Keywords:** ferrihydrite, green rust, microcosm, iron reduction, Fe(II)-catalyzed transformation

## Abstract

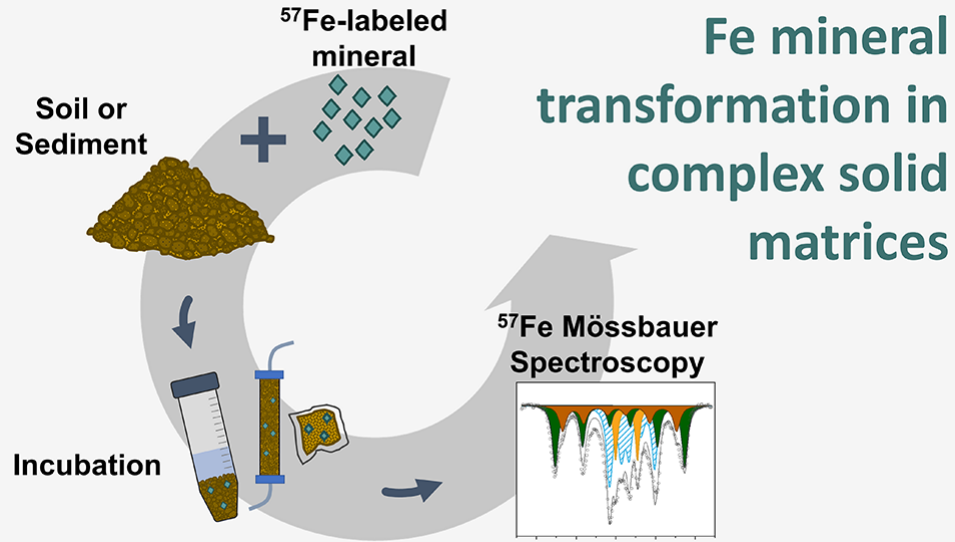

Iron minerals in soils and sediments play important roles in many biogeochemical processes and therefore influence the cycling of major and trace elements and the fate of pollutants in the environment. However, the kinetics and pathways of Fe mineral recrystallization and transformation processes under environmentally relevant conditions are still elusive. Here, we present a novel approach enabling us to follow the transformations of Fe minerals added to soils or sediments in close spatial association with complex solid matrices including other minerals, organic matter, and microorganisms. Minerals enriched with the stable isotope ^57^Fe are mixed with soil or sediment, and changes in Fe speciation are subsequently studied by ^57^Fe Mössbauer spectroscopy, which exclusively detects ^57^Fe. In this study, ^57^Fe-labeled ferrihydrite was synthesized, mixed with four soils differing in chemical and physical properties, and incubated for 12+ weeks under anoxic conditions. Our results reveal that the formation of crystalline Fe(III)(oxyhydr)oxides such as lepidocrocite and goethite was strongly suppressed, and instead formation of a green rust-like phase was observed in all soils. These results contrast those from Fe(II)-catalyzed ferrihydrite transformation experiments, where formation of lepidocrocite, goethite, and/or magnetite often occurs. The presented approach allows control over the composition and crystallinity of the initial Fe mineral, and it can be easily adapted to other experimental setups or Fe minerals. It thus offers great potential for future investigations of Fe mineral transformations *in situ* under environmentally relevant conditions, in both the laboratory and the field.

## Abstract 2

Our new experimental approach using ^57^Fe as a tracer allows investigation of iron mineral transformation processes in complex natural soil or sediment matrices.

## Introduction

In periodically anoxic soil environments, ferric iron (Fe(III)) in minerals and dissolved ferrous iron (Fe(II)) can coexist, and reactions between Fe(III) in minerals and aqueous Fe(II) lead to Fe(II)-catalyzed transformation and recrystallization processes.^1−3^ Additionally, Fe(III) in minerals can react with dissolved S(-II) or be microbially reduced, leading to reductive Fe mineral dissolution and mineral transformations. These processes can change the specific surface area and reactivity of Fe minerals in soils and sediments and thereby affect geochemical processes, including the biogeochemical cycling of elements such as carbon (C), nitrogen (N), and sulfur (S),^4−7^ the incorporation and release of trace elements associated with Fe minerals,^7−10^ and the Fe(II)–Fe(III) mineral-mediated reduction of contaminants.^11,12,43^

Laboratory studies investigating abiotic Fe mineral transformations and recrystallization are often based on synthetic Fe(III) (oxyhydr)oxides reacted with aqueous Fe(II) or S(-II) in buffered aqueous solutions. Such an approach provides maximum control of initial Fe-phase composition and experimental conditions, which allows testing of individual factors influencing mineral transformation pathways and mechanisms. Abiotic model studies have demonstrated that the reaction of weakly crystalline Fe(III) (oxyhydr)oxides with aqueous Fe(II) under anoxic conditions can lead to the formation of more crystalline secondary Fe(III) phases.^13^ Moreover, experiments involving stable Fe isotopes in the aqueous or solid phase have demonstrated rapid electron transfer between adsorbed Fe(II) and structural Fe(III), leading to the exchange of Fe atoms between the aqueous and solid pools.^2,14^ Similarly, biotransformation of Fe minerals has been investigated through laboratory incubations in the presence of bacteria. Such studies have demonstrated that microorganisms can exploit the energy from the oxidation of organic matter using Fe(III) as the terminal electron acceptor during dissimilatory Fe(III) reduction.^15,16^ Biotic and abiotic experiments have further demonstrated that the reaction of Fe(III) minerals with S(-II) leads to the formation of Fe(II) and may form reduced Fe-sulfide minerals.^17−22^

Together, abiotic and biotic laboratory experiments have shed light on possible mechanisms, pathways, and factors controlling Fe mineral transformations. For example, these model studies have shown that the Fe(II)/Fe(III) ratio,^23,24^ S(-II)/Fe(III) ratio,^25^ presence of anions,^1,26^ additional mineral phases,^27,28^ and pH^1,29,30^ are important parameters governing which mineral phases will be formed and the kinetics of transformation. However, in such experiments, it is difficult to capture the full complexity found in nature, such as the natural soil buffering capacity, complex soil biota, presence of other mineral phases and solutes, and pore-scale diffusion processes, to name a few. Such factors together are likely to exert major influences on Fe mineral transformations. However, studies on Fe mineral transformations in soils and sediments are still rare.

Some previous studies have investigated redox-induced transformations of native Fe oxide minerals in soils by exposing the soil materials to repeated redox cycles^31,32^ or by studying and comparing selected soils collected in the field,^31^ analyzing the soil’s Fe speciation by Mössbauer spectroscopy.^31,32^ These studies suggested that the crystallinity of Fe oxide minerals in soils may increase or decrease with repeated redox cycles, depending on the initial Fe oxide crystallinity and water leaching rate.^31^ So far, such studies were limited to soils containing fairly high total Fe contents (>70 g kg^–1^ Fe), in part due to analytical constraints. Laboratory soil incubation experiments were mostly conducted using mixed soil slurries with lower soil-to-solution ratio than in soils under field conditions, which allows purging of the reactors with N_2_ and O_2_ gases to control the redox conditions.^31,32^ Other studies have employed mesh bags filled with Fe minerals and incubated in soil microcosms or in the field.^33−36^ Incubation of mesh bags filled with ferrihydrite in flooded soils led to extensive transformation of ferrihydrite to goethite and lepidocrocite.^34−36^ Recently, one study used gel-based diffusive samplers in the field and demonstrated ferrihydrite sulfidation in a sulfidic tidal flat sediment.^37^ While the studies using mesh bags or diffusive samplers analyzed the transformation of added Fe minerals in conditions that more closely resemble natural systems than in mixed mineral suspensions, they do not facilitate contact of the Fe minerals with microorganisms and other soil components and create local environments at the microscale in which the Fe minerals comprise a much higher fraction of the solids than in the surrounding soil. For example, a recent micro-Raman spectroscopic study demonstrated that the lack of soil contact and diffusion of ferrous Fe from the surrounding soil into the mesh bags filled with ferrihydrite can lead to diffusion processes influencing the mineral transformation pathways.^36^

Here, we propose a novel approach for investigating *in situ* transformations of Fe minerals mixed with soils or sediments, thereby ensuring a close spatial association or direct contact of the Fe mineral with the complex soil or sediment matrix including other minerals, organic matter, and microorganisms. In this approach, we synthesize Fe minerals that are strongly enriched with the stable isotope ^57^Fe, mix the minerals with soil or sediment materials containing much less ^57^Fe, incubate the mineral-enriched soils under chosen conditions, and subsequently use ^57^Fe Mössbauer spectroscopy to investigate the mineral transformation products. Our approach combines the advantage of having maximum control over the initial mineral phase composition and crystallinity with the complexity of studying the mineral transformations in a soil matrix containing other minerals, organic matter, microorganisms, and solutes. Here, we employed ^57^Fe-labeled ferrihydrite as a model Fe mineral and investigated ferrihydrite transformations in microcosm incubations with four soils from vastly differing redox-affected environments to demonstrate the feasibility of the approach. We suggest that this experimental approach can easily be adapted to other setups, both in the laboratory and in the field, opening new avenues to studying Fe mineral transformations in soils and sediments.

## Materials and Methods

We conducted a series of microcosm incubation experiments to test whether our proposed new approach is suitable for investigating Fe mineral transformations in close contact with soils. Ferrihydrite (more information is available in Supporting Information, Section 1.1) was used as the initial ^57^Fe-labeled mineral, and a total of four soils differing in chemical and physical properties were selected for microcosm incubations.

### Soils

In the first step (“main experiment”), we conducted a microcosm incubation experiment with a sandy rice paddy soil from Thailand (herein referred to as “Paddy Soil”). This soil was chosen because a similar soil from the same region demonstrated the potential for Fe reduction and transformation of ferrihydrite into goethite and lepidocrocite in an experiment using mesh bags.^36^ In the second step, an additional microcosm incubation experiment was performed with three soils originating from contrasting environments that experience substantial variations in redox conditions, thereby covering a wide range of soil properties such as texture, pH, Fe content, C content, and trace element concentrations. The soils were collected from an intertidal flat from Germany (referred to as “Intertidal Sediment”), a river floodplain soil from Switzerland (referred to as “Floodplain Soil”), and a clay-rich acid sulfate paddy soil from Thailand (referred to as “Acid Sulfate Soil”). Soils were collected under dry and oxic conditions, and the Intertidal Sediment was collected during low tide when the sediment was drained. All soils were dried at 30 °C, sieved through a 2 mm sieve, and stored in an ambient atmosphere until use. The elemental composition of bulk soils was measured by XRF (XEPOS, Spectro); soil C and N contents were measured by combustion on milled soils (Vario MAX Cube, Elementar, Germany, Table S2), and Mössbauer spectroscopy was used to characterize the native Fe phases in the air-dried soils (Supporting Information, Section 4).

### Soil Microcosm Incubation Experiments

All experiments were carried out in an anoxic glovebox (MBraun, N_2_ atmosphere, <5 ppm O_2_), and all solutions were purged for at least 2 h with N_2_ (99.995% purity) before they were transferred to the glovebox. The soil incubations with the Paddy Soil were performed by adding 15 g of dry Paddy Soil and 65 mg of ^57^Fe-ferrihydrite to 50 mL polypropylene centrifuge tubes (Figure S15). We calculated the ideal amount of ^57^Fe-ferrihydrite to be added to optimize the Mössbauer signal and allow the tracing of ^57^Fe while minimizing the amount of ferrihydrite added to minimize the changes in pore water geochemical conditions or sorption capacity (details in Supporting Information, Section 2.1). In the main experiment, Fe added as ferrihydrite corresponded to 36% of the total Fe in the mixture. The ^57^Fe added as ferrihydrite contributed 96% of the total ^57^Fe in the mixture (Table S1), meaning that the Mössbauer signal will mainly come from the added Fe. To homogenize the spiked soil, we thoroughly mixed the dry solids and kept them in glovebox atmosphere for 1 week to remove sorbed oxygen.

In the process of optimizing the microcosm experiment, we tested multiple conditions. Specifically, we tested the use of background electrolytes, the sealing of the tubes, and tube agitation. Based on the results of the preliminary tests, the use of 0.5 mM CaCl_2_ solution, closing the tubes with Parafilm with caps placed on top but not closed firmly, and not agitating the samples did not change the geochemical conditions in the aqueous phase or the extent or products of ferrihydrite transformation (Supporting Information, Section 5). Therefore, we decided to employ the following conditions in all experiments: inside the glovebox, 15 mL of anoxic 0.5 mM CaCl_2_ was added to the tubes (∼12 mL headwater), and the solids were gently stirred to release any gas bubbles trapped in them. The tubes were sealed with Parafilm with caps placed on top but not closed firmly, wrapped in aluminum foil, and kept still in the glovebox. Sufficient tubes were prepared to enable sacrificial sampling at 1, 2, 4, 8, and 12 weeks (single tubes) and at 16 weeks (tubes sampled in duplicate). Additionally, a set of controls were prepared with soils incubated without the addition of ferrihydrite and were sampled sacrificially at 6 and 12 weeks (tubes sampled in duplicate).

Additional microcosms were prepared using the Intertidal Sediment, the Floodplain Soil, and the Acid Sulfate Soil and incubated under the same conditions to create a reduced soil environment. We calculated the ideal amounts of ^57^Fe-ferrihydrite to be added to each soil (Table S1) based on the percentage of Fe in the soil or sediment (Supporting Information, Section 2.1). For each soil or sediment, four microcosms were prepared: two spiked with ^57^Fe-ferrihydrite and two spiked with ferrihydrite which was not isotopically labeled (natural abundance ^NA^Fe-ferrihydrite). At 4 and 12 weeks, two microcosms were sampled (one with ^57^Fe-ferrihydrite and one with ^NA^Fe-ferrihydrite). For the microcosms with additional soils, the aqueous phase of all samples was analyzed, but only solids from samples spiked with ^57^Fe-ferrihydrite were analyzed with Mössbauer spectroscopy.

### Sampling Procedure

Two to three hours before sampling, oxidation–reduction potential (ORP) and pH were measured in each tube inside the glovebox using a pH (double-junction electrode, 3 M KCl, Metrohm AG, Switzerland) or ORP electrode (Pt electrode combined with an internal Ag/AgCl reference (3 M KCl) electrode; Metrohm AG, Switzerland, Eh = ORP +209 mV). To perform the measurements, the cap and Parafilm were removed, and both electrodes were inserted into the headwater at ∼0.5 cm above solids. Once stabilized (∼5 min for pH, and ∼2 h for ORP), potentials were read, and electrodes were taken out of the headwater. The tube was then closed with a cap, removed from the glovebox, photographed, shaken, and centrifuged at 3000*g* for 25 min. Next, the tube was returned to the glovebox. The supernatant was poured into a syringe, filtered (0.22 μm, nylon, BGB), and separated into three parts. Approximately 1 mL was frozen at −20 °C for ion chromatography (IC) analysis. Then, 3 mL aliquots were acidified with 1 M HCl (Normatom, VWR) to pH 3–4 and stored in glass vials at 4 °C for dissolved organic carbon (DOC) analysis. The remaining filtered aqueous phase was acidified to a final concentration of 0.1 M HCl and stored in plastic vials at 4 °C for major cation content, Fe speciation, and Fe isotope analyses. Details on aqueous phase analyses are reported in the Supporting Information (Section 1.2). The solid sample was kept in the closed tube, brought outside the glovebox, and immediately placed into a flask containing liquid nitrogen for instant freezing. The frozen solids were freeze-dried, immediately returned to the anoxic glovebox, homogenized via manual shaking, and stored in an anoxic atmosphere until analysis with Mössbauer spectroscopy (Supporting Information, Section 1.3).

## Results

### Microcosms with Paddy Soil: Aqueous Phase Characterization

**Figure 1:**
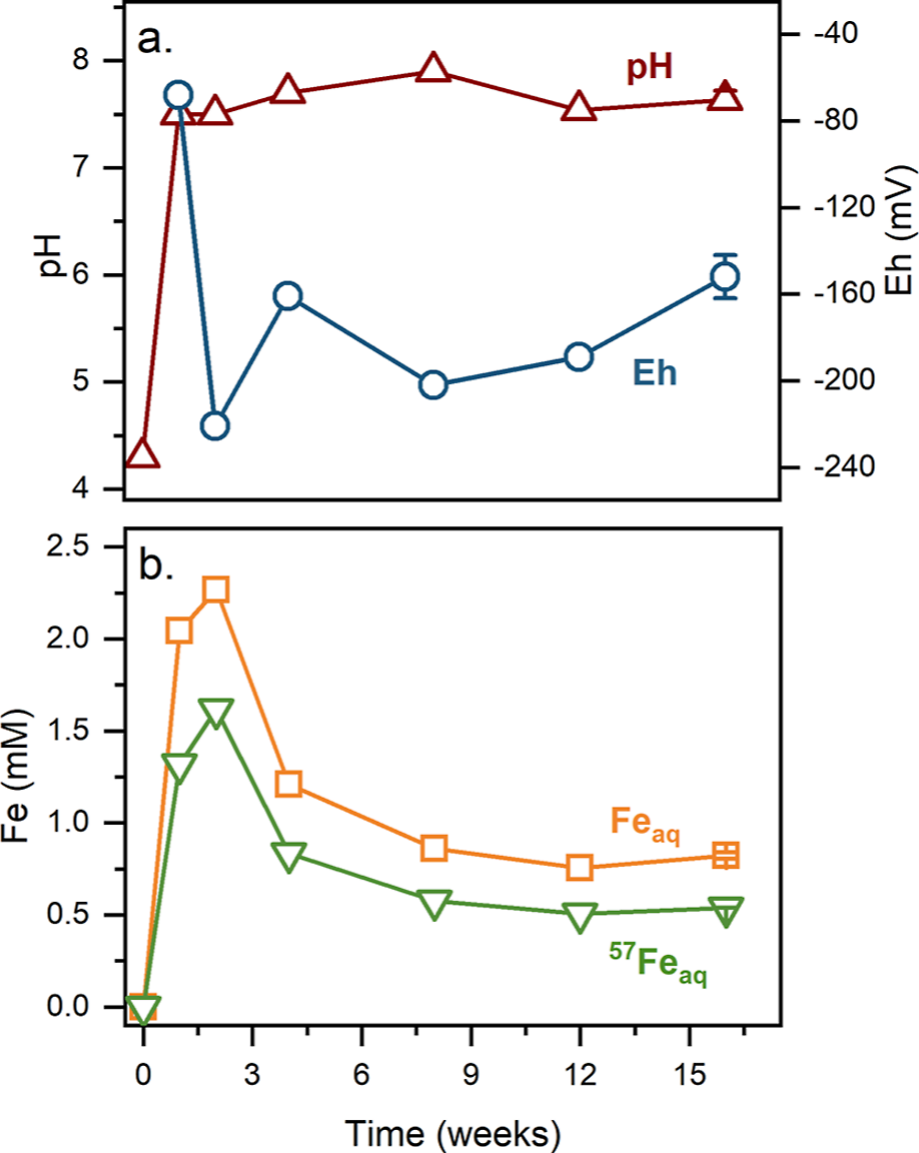
Aqueous phase values of (a) Eh and pH and (b) Fe and ^57^Fe concentrations during 16 weeks of incubation of Paddy Soil spiked with ferrihydrite. Error bars are the range of duplicates of the 16 week samples. Note: The Eh value was not measured at day zero, and the pH value for day zero was measured 3 h after incubation. The Fe concentration was measured with ICP-OES, and ^57^Fe concentration was calculated using the fraction of ^57^Fe in the aqueous phase measured with ICP-MS (Table S5 and Figure S7a).

The temporal developments of Eh, pH, aqueous total Fe, and ^57^Fe concentrations during the 16 week incubation period of the Paddy Soil (main experiment) are shown in Figure 1, and the concentrations of major anions and cations are reported in the Supporting Information (Figure S7). After flooding the soil, the Eh values dropped rapidly to reach −68 mV after 1 week and to near −220 mV after about 2 weeks, after which Eh stabilized at slightly higher values (−150 mV at 16 weeks, Figure 1a). Simultaneously, the pH values increased from 4.3 in the initial 3 h to near 7.6 in the following weeks (Figure 1a). The concentration of DOC in the aqueous phase peaked at 53 mg L^–1^ after 1 week, with a slight decrease during the following weeks (Figure S6c). The concentration of aqueous Fe reached a maximum of 2 mM after 2 weeks, which later stabilized near ∼0.7 mM (Figure 1b). At all time points, aqueous Fe was predominantly Fe(II) (>92%, Table S4). Therefore, for simplicity, aqueous Fe will be referred to as aqueous Fe(II).

To assess whether aqueous Fe(II) was formed by the microbial reduction of added ^57^Fe-ferrihydrite (^57^Fe/Fe_T_ = 0.96) or by the reduction and dissolution of other Fe minerals present in the soil (^57^Fe/Fe_T_ = 0.021), we measured the isotopic composition of aqueous Fe(II). Throughout the incubation period, ^57^Fe/Fe_T_ in the aqueous phase was 0.65 to 0.70, indicating that 62–65% of aqueous Fe(II) originated from the added ^57^Fe-ferrihydrite (see calculations in Supporting Information, Section 2.2). In control microcosms without the addition of ferrihydrite, we observed a higher DOC concentration in the aqueous phase compared to the ferrihydrite-spiked soil (Figure S6), but most other element concentrations were in a similar range (Figure S7).

### Microcosms with Paddy Soil: Mössbauer Spectroscopy

**Figure 2:**
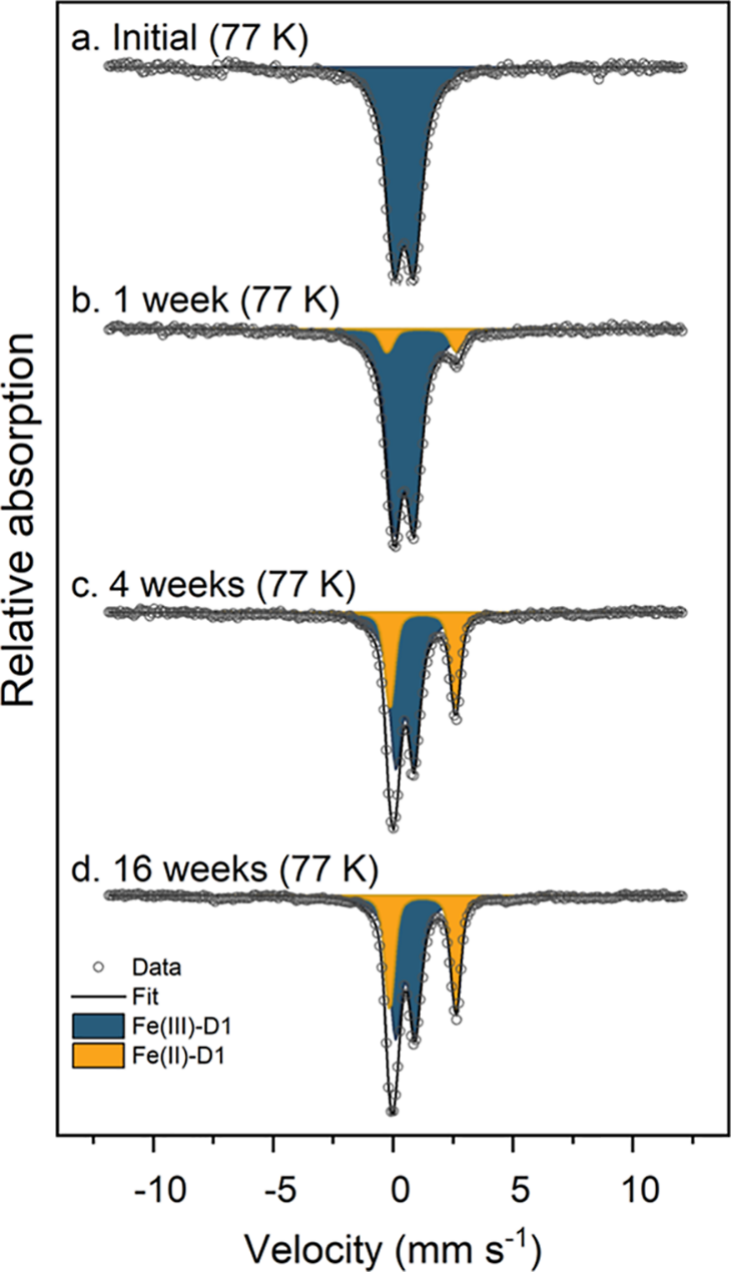
Fitted Mössbauer spectra of the Paddy Soil spiked with ^57^Fe-ferrihydrite, before and after 1, 4, and 16 weeks of incubation. Spectra were collected at 77 K. Fitting parameters are detailed in Table S6. Abbreviations: Fe(III)-D1 = Fe(III) doublet; Fe(II)-D1 = Fe(II) doublet.

In the Paddy Soil spiked with ^57^Fe-labeled ferrihydrite, 96% of the total ^57^Fe, which was detected by Mössbauer spectroscopy, came from the added ^57^Fe-ferrihydrite (Table S1). The Mössbauer spectrum of the initial mixture collected at 77 K revealed a broad doublet with a center shift (CS) of 0.45 mm s^–1^ and a quadrupole splitting (QS) of 1.10 mm s^–1^ (Figure 2a), slightly larger than the values commonly reported in the literature for ferrihydrite (QS ∼ 0.76 mm s^–1^)^38,39^ but still consistent with ferrihydrite starting to undergo magnetic ordering.

One week after the flooding of the Paddy Soil, a second doublet emerged in the 77 K Mössbauer spectra, accounting for 9% of the total solid-phase ^57^Fe. The second doublet required fitting parameters, CS of 1.21 mm s^–1^ and QS of 2.92 mm s^–1^, compatible with the Fe(II) species.^40^ In the following weeks, the incubated solids presented similar spectra, with gradual changes in the fitting parameters such as a slightly larger QS and a larger area fitted as an Fe(II) doublet (Table S6 and Figures 2 and S10). At the end of 16 weeks’ incubation, the Fe(II) doublet accounted for 39% of the total ^57^Fe and stabilized with the fitting parameters of CS of 1.24 mm s^–1^ and QS of 2.76 mm s^–1^. The Fe(III) phase that started as ferrihydrite presented a final fit with a CS of 0.51 mm s^–1^ and QS of 1.03 mm s^–1^, consistent with ferrihydrite but possibly containing some additional Fe(III) phases. The duplicates sampled after 16 weeks of incubation were very similar, suggesting good repeatability of the experiment (Figure S10 and Table S6).

**Figure 3:**
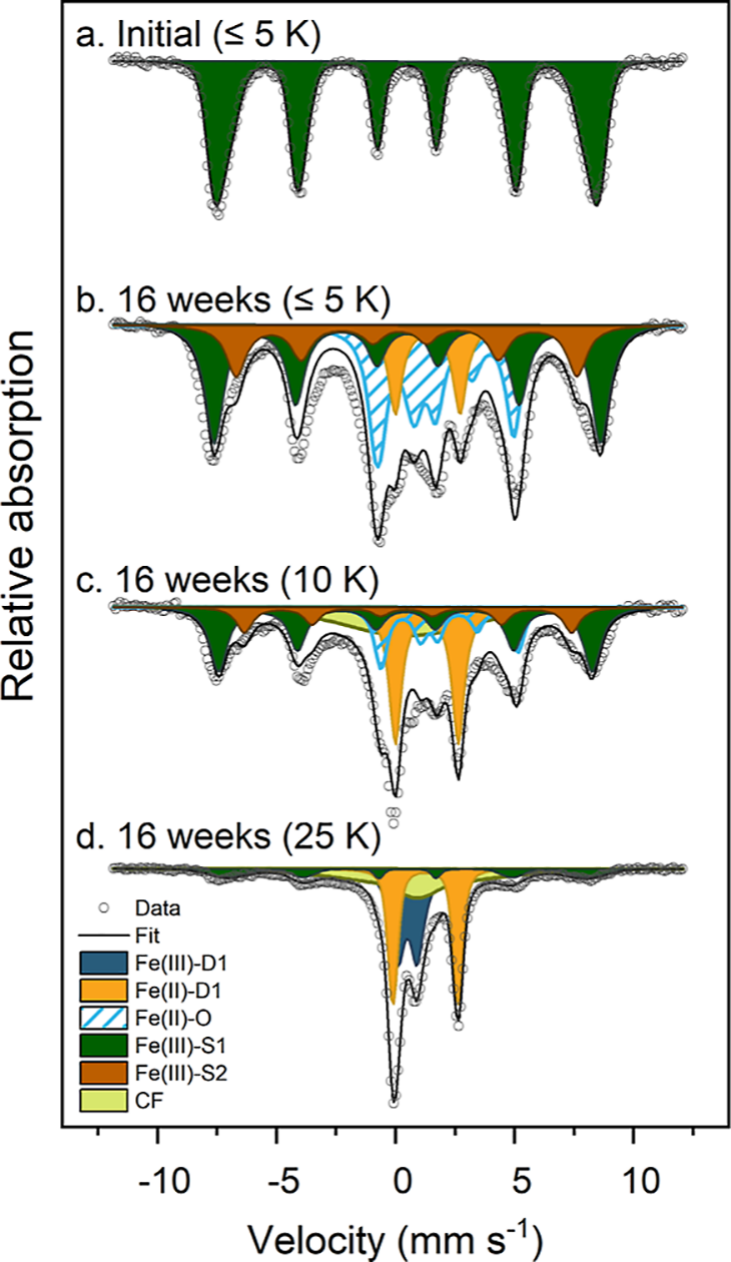
Fitted Mössbauer spectra of the Paddy Soil spiked with ^57^Fe-ferrihydrite before and after 16 weeks of incubation. Spectra were collected at 25, 10, and ≤5 K. Fitting parameters are detailed in Tables S7 and S8. Abbreviations: Fe(III)-D1 = Fe(III) doublet; Fe(II)-D1 = Fe(II) doublet; Fe(II)–O = Fe(II) octet; Fe(III)-S1/2 = Fe(III) sextet; CF = collapsed feature.

While Mössbauer spectra collected at 77 K were good for quantifying the ratio of Fe(II) to Fe(III), they did not allow for a precise identification of the Fe species. To further investigate the nature of the Fe(II) and Fe(III) species formed from ^57^Fe in the added ferrihydrite, we collected additional Mössbauer spectra of the 16 weeks’ sample at a range of temperatures (Figures 3 and S12 and Tables S7 and S8). At temperatures 140, 77, and 45 K, the spectra were dominated by an Fe(II) and an Fe(III) doublet. At 25 and 15 K, an Fe(III) sextet and an undefined collapsed feature started to emerge at the expense of the Fe(III) doublet. At 10 and 5 K, an Fe(II) octet was formed at the expense of the Fe(II) doublet. At the lowest temperature, the spectrum was fitted with an Fe(II) octet, an Fe(II) doublet, and two broad Fe(III) sextets (Fe(III) sextet 1 and 2; fitting parameters are found in Table S8). Spectra collected at 140, 77, 45, 25, and 15 K were fitted using the extended Voigt-based fitting (xVBF) model,^41^ while the spectra collected at 10 and 5 K required the use of the full static Hamiltonian (FSH) fitting model in order to fit the octet.^42^

### Microcosms with Additional Soils

To test the applicability of our approach with soils having contrasting properties, we performed additional experiments with three additional soils (Intertidal Sediment, Floodplain Soil, Acid Sulfate Soil). The results of the aqueous phase characterization of these additional microcosms are shown in Figures S8 and S9. Similar to the Paddy Soil, Eh values recorded through the 12 weeks of incubation dropped, stabilizing at 12 weeks at −239, −187, and −228 mV in the Intertidal Sediment, Floodplain Soil, and Acid Sulfate Soil, respectively (Figure S8a), and the pH values increased and stabilized at pH 8.4 (pH 6.7 at 3 h), at pH 8.6 (pH 7.3 at 3 h), and at pH 7.3 (pH 4.3 at 3 h) (Figure S8b). In all soils, DOC was released into the aqueous phase and peaked (4 weeks) at 83, at 146, and at 148 mg L^–1^ in the Intertidal Sediment, Floodplain Soil, and Acid Sulfate soil, respectively (Figure S8c). The Intertidal Sediment released substantial amounts of Cl^–^ (127.2 mM at 4 weeks) and SO_4_^2–^ (10.9 mM at 4 weeks) into the aqueous phase, while the Acid Sulfate Soil released high concentrations of SO_4_^2–^ (18.0 mM at 4 weeks) and the Floodplain Soil released almost no SO_4_^2–^ (0.002 mM at 4 weeks) (Figure S9). The concentrations of aqueous Fe reached 0.22 mM in the Intertidal Sediment, 0.14 mM in the Floodplain Soil, and 7.7 mM in the Acid Sulfate Soil (Figure S9a). The ratio ^57^Fe/Fe_T_ in the aqueous phase varied significantly among different soils: at 4 weeks reaching 0.67 in the Intertidal Sediment, 0.69 in the Floodplain Soil, and 0.33 in the Acid Sulfate Soil (Table S5). In these incubations, Fe from the spiked ferrihydrite accounted for 24, 20, and 12% of the total Fe in the solid phase, respectively (Table S1).

**Figure 4:**
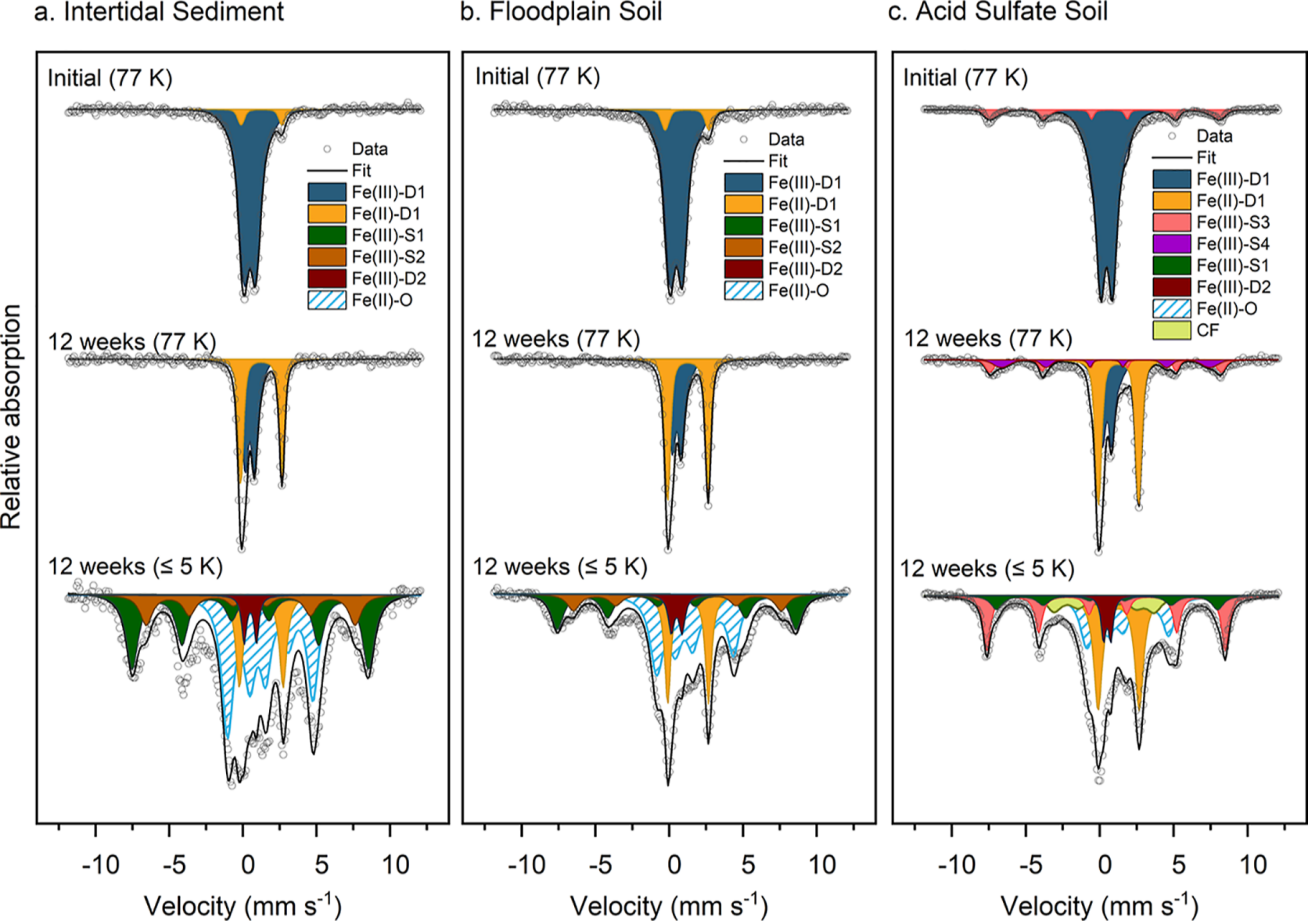
Fitted Mössbauer spectra of (a) Intertidal Sediment, (b) Floodplain Soil, and (c) Acid Sulfate Soil spiked with ^57^Fe-ferrihydrite, before and after 12 weeks of incubation. Spectra were collected at 77 and ≤5 K. Fitting parameters are detailed in Tables S9 and S10. Abbreviations: Fe(III)-D1/2 = Fe(III) doublet; Fe(II)-D1 = Fe(II) doublet; Fe(III)-S1/2/3/4 = Fe(III) sextet, Fe(II)–O = Fe(II) octet; CF = collapsed feature.

The 77 K Mössbauer spectra of the three soils spiked with ^57^Fe-labeled ferrihydrite before incubation were again dominated by a broad doublet (CS = 0.46–0.48 mm s^–1^ and QS = 1.17–1.26 mm s^–1^) (Figure 4), similar to the ferrihydrite component observed in the mixture with the Paddy Soil. The spectra of the Intertidal Sediment and the Floodplain Soil had an additional small Fe(II) doublet which represented 6 and 8% of the total ^57^Fe, respectively. In contrast, the Acid Sulfate Soil exhibited an additional sextet which represented 12% of the total ^57^Fe. For these experiments, the percentage of ^57^Fe coming from the added ferrihydrite accounted for 93, 91, and 85% of the total solid ^57^Fe in the mixtures with the Intertidal Sediment, the Floodplain Soil, and the Acid Sulfate Soil, respectively (Table S1). Therefore, the additional doublets and sextets observed are the likely features from the soils (Figure S4) that are sufficiently distinct from the added ferrihydrite. This was confirmed with the 5 K Mössbauer spectra of initial samples, which also contain distinct Fe(III) doublets and Fe(III) sextets (Figure S13). After 12 weeks of incubation, the 77 K Mössbauer spectra of the solids revealed the formation of an Fe(II) doublet with fitting parameters similar to those found in the incubation with the Paddy Soil (CS = 1.21–1.25 mm s^–1^, QS = 2.72–2.80 mm s^–1^, Figure 4, Table S9). The spectrum of the Acid Sulfate Soil also presented more spectral area as sextets than the initial sample (22% compared to 12%).

The 5 K Mössbauer spectra of samples incubated for 12 weeks revealed the formation of octets similar to those found for the incubations of the Paddy Soil (Table S10), with an Fe(II) octet, Fe(II) doublet, and two broad sextets (Figure 4). Additionally, all three soils required an Fe(III) doublet in the 5 K spectra, a feature likely from the soils observed in the mixture before incubation (Figure S13). The Mössbauer features of the Intertidal Sediment were similar to those found in the incubation experiments of the Paddy Soil, with 41% of the spectral area fitted as an octet and 8% as a paramagnetic Fe(II) doublet. In the Floodplain Soil and the Acid Sulfate Soil, higher fractions of Fe(II) remained as a paramagnetic doublet instead of ordering into an octet (18 and 26%, respectively). Additionally, the Acid Sulfate Soil showed an additional sextet, along with a collapsed feature. No significant differences were observed between the 4 week and 12 week Mössbauer spectra of the additional soils, suggesting most changes occurred during the first 4 weeks of incubation (Figure S14).

## Discussion

### Transformation of Ferrihydrite in the Paddy Soil

Upon the flooding of the Paddy Soil microcosms, the Eh values decreased, reflecting reducing conditions in which Fe(III) (oxyhydr)oxides can serve as terminal electron acceptors for dissimilatory Fe-reducing bacteria, forming reduced Fe species.^43^ The microbial reduction of Fe(III) led to an increase of the pH value (Figure 1a) as a result of the proton consumption by reductive dissolution of Fe(III) (oxyhydr)oxides^44^ and, ultimately, the increase of Fe(II) in the aqueous phase (Figure 1b). In the second half of the incubation process, we observed a decrease in the aqueous Fe(II) concentration, suggesting the formation of Fe(II) or mixed-valence solid phases, or sorption of Fe(II) onto solid phases. For all incubated samples, the 77 K Mössbauer spectra of reacted solids were dominated by an Fe(II) and an Fe(III) doublet, both with non-unique fitting values, as evidenced by the graphic summary (Figure S11) of values and commonly reported fitting values for siderite,^45^ vivianite,^46,47^ ferrous hydroxide,^48^ Fe(II) sorbed onto Fe oxides,^13^ and green rust.^49^ Despite the complex spectra which are challenging to fit, we can conclude that the sample did not contain detectable amounts of magnetite (sextet at 77 K),^50^ mackinawite (singlet or collapsed feature at 77 K),^51,52^ or greigite and intermediate FeS_x_ phases (sextets with a low hyperfine field).^51^ However, we cannot eliminate the possibility of pyrite formation since the typical fitting parameters of pyrite are too similar to those of an Fe(III) doublet at 77 K.^51^ The detailed temperature profile of the Paddy Soil incubated for 16 weeks provided additional insights into the Fe phases, including an octet phase that developed at lower temperatures. Fitting the octet required the use of a full static Hamiltonian site analysis (FSH), which revealed parameters similar to those reported for green rust.^53−55^ Furthermore, our temperature profile data revealed that the Fe(II) octet only started to form below 15 K, and it is possibly not fully ordered at 5 K (Figures 3 and S12). Such information precludes its interpretation as siderite (Néel temperature ∼ 37 K^56^) or ferrous hydroxide (Néel temperature ∼ 34 K^57^). Instead, the octet ordering temperature is similar to green rust (Néel temperature ∼ 5.2 K^54^) and vivianite (Néel temperature ∼ 12 K^58^). However, the parameters of our 77 K Fe(II) doublet did not contain the characteristically high QS of vivianite.^46^ Taken together, the 77 K fit of the doublet, the 5 K fitting parameters of the octet, and the ordering temperature observed in the temperature profile support our interpretation that the solid Fe(II) phase formed upon the incubation of ferrihydrite in the Paddy Soil is a green rust-like phase.

Green rust has a theoretical Fe(II)/Fe(III) stoichiometry of 2:1. Considering that 32% of the solid ^57^Fe was found as Fe(II) ordered as a green rust-like octet (Table S8), we estimate that up to 48% of ^57^Fe was in the green rust-like phase. However, in the 5 K spectrum, 11% of ^57^Fe remained as an Fe(II) doublet. While this Fe(II) doublet could be Fe(II) sorbed onto Fe minerals,^13,59,60^ or Fe(II) in clays,^61^ it could also be green rust before the complete ordering of the octet that occurs at ∼1.4 K.^54^ In addition, isomorphic substitution with Mg^2+^ or Mn^3+^ could further complicate the determination of the stoichiometry of green rust phases with Mössbauer,^62^ although the aqueous concentrations of Mg^2+^ or Mn^3+^ in our incubation with Paddy Soil are 1 order of magnitude smaller than those of Fe (Figure S7). Still, here, we will refrain from quantifying the green rust-like fraction. Yet, it is safe to state that at least 32% of the total ^57^Fe in the solids were present as the Fe(II) component of the green rust-like phase. The most common types of green rusts (GR) are hydroxy sulfate (GR(SO_4_^2–^)), hydroxy carbonate (GR(CO_3_^2–^)), and hydroxy chloride (GR(Cl^–^)) green rusts.^75^ While the presence of different anions causes a slight difference in the Mössbauer fitting parameters of a 77 K spectrum,^63^ such differences are likely hidden in our complex spectra with multiple Fe phases.

In addition to the octet, the FSH fit of the 16 week 5 K spectra revealed the presence of two broad sextets (Fe(III) sextet 1 and 2). However, the FSH fitting routine cannot capture the broadness of the Fe(III) sextets, not even for our initial ferrihydrite sample (Figure S3). Based on the fitting parameters for the Fe(III) sextet 1 (CS = 0.50 mm s^–1^, quadrupole shift (ε) = 0.00 mm s^–1^, and H = 50.28 T), this phase could be considered to be either ferrihydrite^64,65^ or the Fe(III) fraction of green rust.^54^ Considering that the Fe(III) sextet 1 starts to order before 25 K, this phase is likely to be ferrihydrite, since the Fe(III) component of green rust orders at ∼7 K.^54^ The Fe(III) sextet 2 might be a spectral area that could have been fit by adding more broadness to the Fe(III) sextet 1 (limitation of the FSH fitting routine), but it also has fitting parameters compatible with lepidocrocite.^66^ Therefore, to fit the green rust-like octet, the use of the FSH model was essential, which interfered with our ability to identify phases in the Fe(III) fraction. We thus suggest that the sample is composed of a mixture of ferrihydrite with the Fe(III) fraction of green rust and possibly some lepidocrocite. Confirming the precise identity of the Fe phases that are mixed in soils is challenging since the low amount of Fe in the mixture and the presence of primary silicate minerals prevent the use of other analytical methods, such as XRD and Fe K-edge EXAFS.

Investigating the transformation of ferrihydrite in the Paddy Soil using our approach with ^57^Fe-labeled minerals and Mössbauer spectroscopy revealed no (or minimal) formation of crystalline Fe(III) (oxyhydr)oxides. Such results contrast investigations that employed mineral suspensions spiked with Fe(II),^23,28,67−70^ dissimilatory Fe reduction of ferrihydrite under advective flow,^71^ or incubations in paddy soils employing mesh bags^36^ in which the formation of lepidocrocite, goethite, and/or magnetite was observed as major fractions. Thus, our results indicate that the influence of close contact with the soil matrix can result in different mineral transformation processes. For example, the decrease in DOC and Si aqueous concentration in the incubations with the added ferrihydrite (Figures S6 and S7) suggests that the ferrihydrite sorbed organic matter and silicate, which have been shown to hinder the transformation of ferrihydrite into more crystalline Fe(III) (oxyhydr)oxides.^38,40,72,73^ A similar phenomenon was recently observed in a study with ferrihydrite filled into mesh bags and incubated flooded paddy soil microcosms, leading to an outer rim of apparently unreacted ferrihydrite, which was attributed to the contact with the dissolved components of pore water such as phosphorus, silicate, and/or organic matter.^36^

Instead of crystalline Fe(III) (oxyhydr)oxides, as often observed in ferrihydrite suspensions spiked with aqueous Fe(II), our results revealed the formation of a green rust-like phase. Formation of green rust via mineralogical transformation of Fe(III) (oxyhydr)oxides requires an Fe(II)/Fe(III) ratio of 2:1^74,75^ and has been shown to occur via the slow addition of Fe(II) to ferrihydrite.^76^ The formation of green rust has also been reported via biotransformation of ferrihydrite and lepidocrocite by dissimilatory iron-reducing bacteria (DIRB),^46,77−79^ and *in situ* studies have identified green rusts in water-logged soils.^63,80^ The formation of green rust in our soil microcosms may have been favored by locally high Fe(II) concentrations in contact with the added ferrihydrite at near-neutral pH, both induced by microbial Fe reduction. The formation of other mixed-valence Fe minerals such as magnetite and crystalline Fe(III) (oxyhydr)oxide minerals such as goethite and lepidocrocite may have been hindered by the presence of anionic solutes such as phosphate,^81,82^ silicate,^73,81,83^ or DOC,^38,72,84^ further favoring the formation of green rust. While the formation of green rust must have been favored by the specific geochemical conditions established in the non-mixed and anoxic soil microcosms, such conditions can also develop in flooded or water-logged soils, which are also non-mixed and diffusion-limited porous media with a high solid-to-solution ratio. In contrast, green rust formation may be less favorable in more dilute (lower solid-to-solution ratio) and mixed soil slurry experiments. Although green rust was observed in all soils here, it may not always form in soil microcosms. However, the identification of green rust as a transformation product of ferrihydrite was only possible in this study due to our experimental approach. Future studies could further investigate the specific soil conditions required to form green rust under laboratory or field conditions. For example, green rust is not commonly detected in paddy soils under field conditions, presumably because of its high sensitivity to oxidation and other analytical limitations, but geochemical speciation calculations have revealed that some paddy soil solutions may be oversaturated with respect to green rusts.^85^

### Application to Other Soils

To verify the applicability of our approach with different soils, we incubated ^57^Fe-ferrihydrite with three other soils from contrasting environments. Since each one of these soils contained its own unique set of geochemical characteristics, the aqueous phase also revealed contrasting parameters (Figures S8 and S9). Porewater in microcosms with the Intertidal Sediment contained higher pH, K^+^, Mg^2+^, Na^+^, Cl^–^, Br^–^, and SO_4_^2-^, which is consistent with seawater influence and thus typical for coastal sediments.^86^ Microcosms with the Floodplain Soil showed DOC concentrations that were comparable to the observations in the field, where water-extractable organic carbon was analyzed in similar floodplain soils.^87^ Lastly, the Acid Sulfate Soil led to an aqueous phase with high Fe and SO_4_^2–^, as observed in such environments.^88−91^

Despite significant differences between the three soils in the composition of aqueous and solid phases, the transformation of ferrihydrite into a green rust-like phase was observed in all soils tested in this study (Figure 4). ^57^Fe-ferrihydrite incubated in the Intertidal Sediment and Floodplain Soil led to similar ferrihydrite transformation products as found in the Paddy Soil, with the addition of an Fe(III) doublet, likely to be Fe(III) surface-complexed or Fe(III) in silicates^32^ from the soils, but the possibility of pyrite^51^ formation cannot be excluded. However, ^57^Fe-ferrihydrite incubation in the Acid Sulfate Soil formed additional mineral transformation products. The 5 K Mössbauer spectra of the 12-weeks incubated ^57^Fe-ferrihydrite in the Acid Sulfate Soil reveals a collapsed feature that we cannot identify based on its fitting parameters (Table S10). Additionally, the 5 K spectrum reveals two sextets, one from the initial ferrihydrite (Fe(III)–S1, likely mixed with the Fe(III) fraction of green rust, as seen in the ferrihydrite incubated in the Paddy Soil) (Figure 4). The second sextet (Fe(III)–S3) has Mössbauer fitting parameters similar to goethite,^28^ nanogoethite,^92^ and more crystalline ferrihydrite^64^ (such as six-line ferrihydrite) and likely consists of their mixture. However, the 5 K Mössbauer spectra of jarosite and schwertmannite are hard to differentiate from goethite/ferrihydrite.^93^ Therefore, while we speculate that the sextet in the 5 K spectra of raw soils and incubated samples (Fe(III)–S3) is (nano)goethite/ferrihydrite, additional techniques would be required to confirm our interpretation. Nonetheless, the 77 K spectra of ferrihydrite incubated in the Acid Sulfate Soil lacks features commonly associated with magnetite, mackinawite,^51,52^ greigite, and intermediate FeS_x_ phases.^51^ Instead, it displays an increase in the area observed as sextets (22% compared to 12% in the initial sample), suggesting the formation of more goethite from ^57^Fe resulting from the reductive dissolution of ferrihydrite. Geochemical conditions such as lower DOC, the presence of SO_4_^2-^, and lower pH^1^ likely favored the transformation of ferrihydrite into goethite in Acid Sulfate Soils. However, other possible explanations include that ^57^Fe reduced from ferrihydrite underwent atom exchange with goethite in the soil,^94^ which became enriched in ^57^Fe and therefore more visible in the Mössbauer spectra, or that the presence of a substantial amount (Table S3) of coexisting goethite in the Acid Sulfate Soil promoted the Fe(II)-catalyzed transformation of ferrihydrite to goethite.^28^

Overall, the incubation of ferrihydrite incorporated into the Paddy Soil investigated in the main experiment, or with the Intertidal Sediment, the Floodplain Soil, and the Acid Sulfate Soil all formed a green rust-like phase during soil flooding (at least 26–41% of ^57^Fe in solid phase). Future studies could investigate if the formation of green rust is also favorable in experimental setups that include advective flow resulting in more mixing at the pore scale. In any case, we can state that the labeling of the studied mineral with ^57^Fe and the detection by ^57^Fe Mössbauer spectroscopy leads to a small increase in total Fe but considerable increases in ^57^Fe (for our Paddy Soil, 52 and 2500%, respectively), allowing us to observe a small fraction of solids in the soils (<0.5% of solids; ∼10% of total Fe atoms) that would likely not be distinguishable using most other techniques (e.g., XRD and X-ray absorption spectroscopy). With our new approach, we have demonstrated that the formation of green rust-like phases is possible in all these soil environments.

### Environmental Implications

By synthesizing ^57^Fe-labeled mineral phases and mixing them with natural soils, our method allowed us to investigate the transformation of ferrihydrite in close spatial association or direct contact with soils. Because our approach with ^57^Fe-labeled Fe minerals allowed us to observe the ferrihydrite transformation products in detail with Mössbauer spectroscopy, we observed the formation of a green rust-like phase, an unusual product in studies that have investigated Fe(II)-catalyzed ferrihydrite transformation in mixed suspensions. As a mixed-valence Fe mineral, green rust might play a crucial role in the biogeochemical cycling of metals and mobility and redox transformation of organic and inorganic pollutants. The formation of a green rust-like phase in all four soils that we tested suggests that such a phase may be more widespread than previously thought, especially in conditions that favor the buildup of locally high Fe(II) concentrations formed during microbial respiration involving Fe(III) minerals as electron acceptors.

More importantly, our study demonstrated that spiking soils and sediments with ^57^Fe-labeled minerals and using Mössbauer spectroscopy to follow mineral transformation is a suitable approach for investigating *in situ* Fe mineral transformations in soils and sediments with different chemical and physical properties. Because it allows for the complete incorporation of the Fe mineral phase into the soil matrix, our approach provides a better analogue to nature than experimental designs such as mineral incubations with mesh bags or gel-based diffusive samplers, which create a spatial separation between the microbially active soil and the Fe minerals studied.^36,37^ While our approach does not investigate the fate of the native Fe species in the soil, adding a synthesized Fe mineral to the soil allows for control over the composition and crystallinity of the initial Fe mineral phase of interest, which opens new avenues for investigating Fe mineral transformation processes under environmentally relevant conditions. Furthermore, the combination of labeled ^57^Fe and ^57^Fe Mössbauer spectroscopy allows for Fe mineral spikes substantially smaller than that using naturally abundant Fe, limiting changes in the geochemical conditions and reducing Mössbauer measurement times. While ferrihydrite was used as the initial ^57^Fe-labeled mineral, this approach can be easily adapted to study other Fe minerals, which may similarly be synthesized from ^57^Fe. Furthermore, while our experiments were conducted with flooded soil microcosms, the new approach using ^57^Fe-labeled minerals mixed into soils or sediments combined with Mössbauer spectroscopy can be easily adapted to other experimental setups including laboratory and field incubation studies.

## Supporting Information Available

Details on experimental and analytical methods; characterization of ferrihydrite and soils by XRD and/or Mössbauer spectroscopy; additional tests of the microcosm setup for robustness; aqueous phase analysis results; Mössbauer spectra and fitting parameters for soil incubation experiments; and photographic records of the samples. (PDF)
