# Supplementary material for: A New Approach for Investigating Iron Mineral Transformations in Soils and Sediments Using 57Fe-Labeled Minerals and 57Fe Mössbauer Spectroscopy
Source: Environ Sci Technol. 2023 Jun 26;57(27):10008–18. doi: 10.1021/acs.est.3c00434 (PMC10339716; doi:10.1021/acs.est.3c00434)
Supplement: Supplementary file 1 — es3c00434_si_001.pdf [file es3c00434_si_001.pdf]

Supplementary Information:

‘A new approach for investigating iron mineral transformations in soils and sediments using ^{57}Fe-labeled minerals and ^{57}Fe Mössbauer spectroscopy’

Luiza Notini[1], Katrin Schulz[1], L. Joëlle Kubeneck[1], Andrew R. C. Grigg[1], Katherine A. Rothwell[1], Giulia Fantappiè[1], Laurel K. ThomasArrigo[1], Ruben Kretzschmar[1*]

[1]Soil Chemistry Group, Institute of Biogeochemistry and Pollutant Dynamics, Department of Environmental Systems Science, ETH Zurich, CHN, Universitätstrasse 16, CH-8092 Zurich, Switzerland.

**Corresponding author: Tel.: +41 44 633 60 03, E-mail:* ruben.kretzschmar@env.ethz.ch; *CHN F 23.1, Universitätstrasse 16, 8092 Zürich, Switzerland*

(35 pages, 19 figures, 10 tables)

Table of contents

1 ADDITIONAL METHODS 3
1.1 Ferrihydrite synthesis 3
1.2 Aqueous phase analysis 3
1.3 Mössbauer spectroscopy 3
1.4 Powder X-ray diffraction 4
1.5 Summary of experimental conditions 5
2 CALCULATIONS 6
2.1 Calculation of the amount of ^{57}Fe-ferrihydrite needed 6
2.2 Calculation of reduction of added ferrihydrite 7
3 FERRIHYDRITE CHARACTERIZATION 8

4 SOILS 9
4.1 Additional information and characterization of soils 9
4.2 Mössbauer spectroscopy of soils 10
4.2.1 Paddy Soil 12
4.2.2 Intertidal Sediment 12
4.2.3 Floodplain Soil 12
4.2.4 Acid Sulfate Soil 12
5 ROBUSTNESS OF THE MICROCOSM EXPERIMENT 14
6 AQUEOUS PHASE ANALYSIS 17
6.1 Aqueous Fe(II) to Fe total (FeT) ratio 17
7 MÖSSBAUER SPECTROSCOPY 23
7.1 Paddy Soil incubation 23
7.2 Paddy Soil incubation – temperature profile 25
7.3 Additional soils incubation 28
8 PHOTOGRAPHIC RECORDS 31
9 REFERENCES 34

1 ADDITIONAL METHODS

1.1 Ferrihydrite synthesis

Ferrihydrite was prepared from ^{57}Fe(0) metal powder (Isoflex, 95.5% isotope purity, henceforth known as ^{57}Fe-ferrihydrite) or ^{NA}Fe(0) metal powder (≥ 99.5 %, natural abundance Fe, henceforth known as ^{NA}Fe-ferrihydrite). For the synthesis of ferrihydrite, 300 mg of Fe(0) was dissolved in 30 mL of 2 M HCl (Normatrom, VWR) to obtain an Fe(II) stock solution. This solution was oxidized with excess H_2O_2 (35%, Merck) and subsequently filtered (0.22 µm, nylon, BGB). Then, the pH was raised to pH 7.5 ± 0.5 by dropwise addition of 1 M KOH (MQ500, Merck) under constant stirring. The resulting precipitates were centrifuged at 3500 *g* for 25 minutes and washed with ultrapure water (UPW, Milli-Q, >18 MΩ·cm) multiple times until the supernatant conductivity was <100 µS cm^{-1}. Then, the washed solids were resuspended in 50 mL UPW. Finally, the suspension was shock-frozen by dropwise injection into liquid N_2, freeze-dried, gently homogenized with a mortar and pestle, and stored in brown glass bottles in a desiccator until use. Powder X-ray diffraction (XRD, see details below) patterns revealed that ferrihydrite (2-line) was the only mineral found in the solids (**Figure S2**).

1.2 Aqueous phase analysis

Frozen aqueous phase samples were thawed and analyzed for Cl^-, Br^-, F^-, and SO_4^{2-} using ion chromatography (IC, Metrohm 940 Professional IC Vario). The dissolved organic carbon (DOC) samples were analyzed with a total organic carbon analyzer (DIMATOC 2000, DIMATEC). Aqueous concentrations of Fe(II) and total Fe were determined using the 1,10-phenanthroline method[1] and used to calculate the percentage of Fe(II) in the aqueous phase. Concentrations of Al, Ca, Fe, K, Mg, Mn, Na, S, and Si were measured with inductively coupled plasma optical emission spectrometry (ICP-OES, Agilent 5100). For Fe isotope analysis, samples were then diluted to 100 ppb Fe and their Fe isotopic composition was determined via inductively coupled plasma-mass spectrometry (ICP-MS, Agilent 8800 Triple Quad) in reaction cell mode with an H_2(g) flow rate of 7 mL min^{-1} to remove argide polyatomic interferences. Fe isotopic composition results were reported as $f\,^{57}$Fe being the counts of the isotope ^{57}Fe divided by the sum of the counts of isotopes ^{54}Fe, ^{56}Fe, ^{57}Fe, and ^{58}Fe, as used in other works.[2, 3]

1.3 Mössbauer spectroscopy

Solid samples were analyzed with Mössbauer spectroscopy in transmission mode using a constant acceleration drive system and a ^{57}Co source in a standard setup (WissEl,

Wissenschaftliche Elektronik GmbH) equipped with a closed-cycle He cryostat (SHI-850, Janis Research Co.) at 77 K and ≤ 5 K. The sample of Paddy Soil spiked with ^{57}Fe-ferrihydrite incubated for 16 weeks was additionally analyzed at 140, 45, 25, and 15 K to support phase identification. Samples were prepared inside the glovebox by sealing ~100-150 mg of solids between two pieces of Kapton polyimide tape. The spectra were fit using the software Recoil (University of Ottawa, Canada) by applying an extended Voigt-Based fitting (xVBF) routine[4, 5] or Full Static Hamiltonian (FSH) fitting routine.[6] The velocity scale was calibrated using a 7 μm thick α-Fe(0) at room temperature. The half-width at half-maximum was fixed to 0.135 mm s^{-1}, the value of the inner line broadening of the calibration foil. All Mössbauer data is presented in relative absorption, where data is normalized between the maximum absorbance and the average of minimum absorption.

1.4 Powder X-ray diffraction

Powder X-ray diffraction (D8 Advance, Bruker) measurement was performed on the initial ferrihydrite. Ferrihydrite was resuspended in ethanol, transferred onto polished Si wafers without XRD background (711 cut, Sil'tronix Silicon Technologies, France), and allowed to dry in ambient air. The wafer was measured between 10° and 70° 2θ with a step size of 0.02° 2θ for 10 s at each step. The sample was analyzed in Bragg–Brentano geometry using Cu-Kα radiation (65.5% $K\alpha_1$, λ=1.540596 Å and 34.5% $K\alpha_2$, λ=1.544493 Å) at 40 kV/40 mA and a high-resolution energy-dispersive 1D detector (LYNXEYE) D8 Advance, Bruker.

1.5 Summary of experimental conditions

Table S1. Summary of experimental conditions and calculations of ^{57}Fe addition.

Soil/Sed. (15 g)	Aqueous phase (15 mL)	Tube closing system	Agitation? (Y/N)	Note	Analysis	Fe in soil/sed. (wt. %)	Mass ^{57}Fe-ferrihydrite added (mg) / 15 g soil	Mass ^{NA}Fe-ferrihydrite added (mg) / 15 g soil	^{57}Fe from added Fh (%)	Fe_{Fh}/Fe_T x100 (%)	Increase in Fe content after addition of Fh (%)
**Paddy Soil**	0.5 mM $CaCl_2$	Parafilm	N	Main exp.	Aqueous + MB	0.55	65	0	96	34.2	52
**Paddy Soil**	UPW water	Parafilm	N	UPW test	Aqueous + MB	0.55	65	0	96	34.2	52
**Paddy Soil**	0.5 mM $CaCl_2$	Closed Cap	N	Closed Cap test	Aqueous + MB	0.55	65	0	96	34.2	52
**Paddy Soil**	0.5 mM $CaCl_2$	Closed Cap	Y	Agitated test	Aqueous + MB	0.55	65	0	96	34.2	52
**Paddy Soil**	0.5 mM $CaCl_2$	Parafilm	N	No Fh Control	Aqueous	0.55	0	0	0	-	0
**Intertidal Sediment**	0.5 mM $CaCl_2$	Parafilm	N	Additional soils	Aqueous + MB	0.92	65	0	93	23.7	31
**Intertidal Sediment**	0.5 mM $CaCl_2$	Parafilm	N	Additional soils	Aqueous	0.92	0	65	0	23.7	31
**Floodplain Soil**	0.5 mM $CaCl_2$	Parafilm	N	Additional soils	Aqueous + MB	1.15	65	0	91	19.9	25
**Floodplain Soil**	0.5 mM $CaCl_2$	Parafilm	N	Additional soils	Aqueous	1.15	0	65	0	19.9	25
**Acid Sulfate Soil**	0.5 mM $CaCl_2$	Parafilm	N	Additional soils	Aqueous + MB	3.8	115	0	85	11.8	13
**Acid Sulfate Soil**	0.5 mM $CaCl_2$	Parafilm	N	Additional soils	Aqueous	3.8	0	115	0	11.8	13

Abbreviations: Sed.= Sediments, Exp. = Experiment, MB = Mössbauer Spectroscopy,
$Fe_{Fh}/Fe_{T:}$ Percentage of Fe in ferrihydrite added compared to the total Fe in the mixture of soil/sediment and ferrihydrite.

2 CALCULATIONS

2.1 Calculation of the amount of ^{57}Fe-ferrihydrite needed

In this study, we tried to minimize the addition of ^{57}Fe-ferrihydrite to soils to allow for a more realistic Fe concentration in the soil and less disturbance in the soil's sorption capacity. The amount of ^{57}Fe-ferrihydrite added to each soil was chosen to ensure that at least 85% of all ^{57}Fe in the mixture was added as synthetic ferrihydrite (red shaded area in **Figure S1**), facilitating the tracing of the added Fh. We also considered that the estimated sample mass in the Mössbauer sample holder (∅ = 13 mm) should not be larger than 120 mg (blue shaded area in **Figure S1**), minimizing the instrument time needed for a good Mössbauer spectrum. Depending on the amount of Fe naturally in the soil, we plotted the estimated sample mass required in the Mössbauer sample holder and the percentage of the spectrum from ^{57}Fe-ferrihydrite as a function of added mass of ^{57}Fe per gram of soil in the mixture. Then we picked the minimum value that fulfills both conditions. **Figure S1** has examples of graphs calculated for soils with 0.5, 1, 3.8, and 6% Fe.

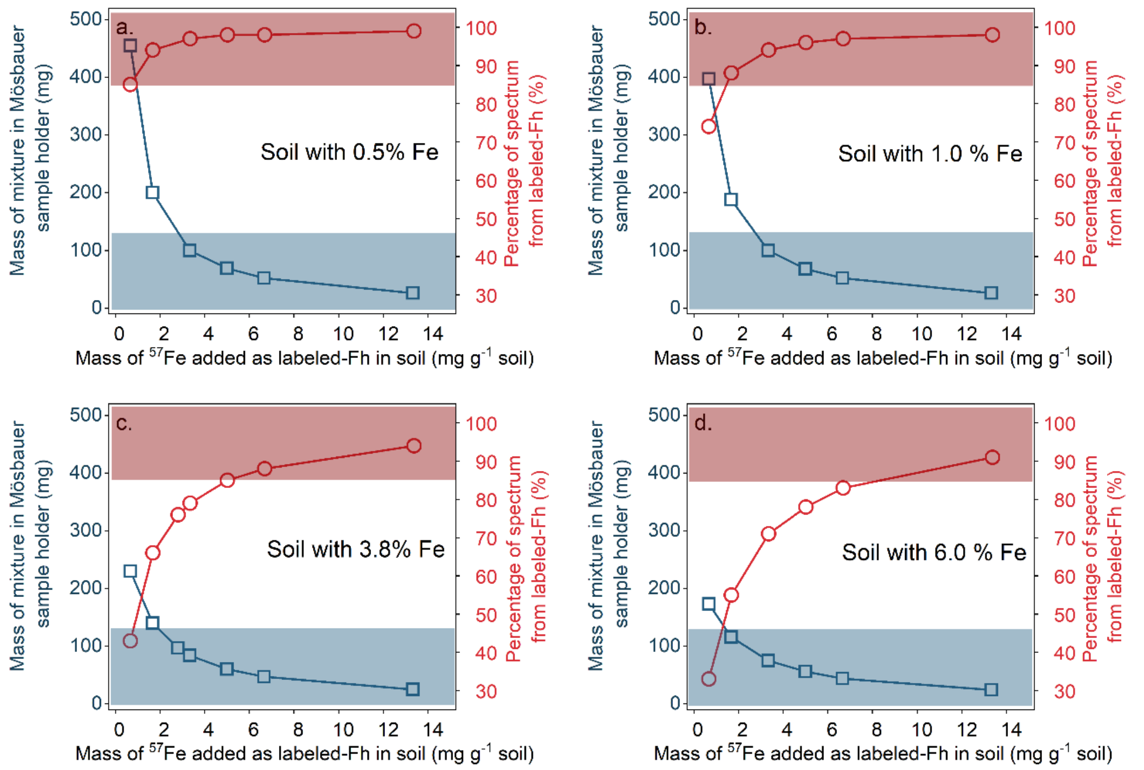

Figure S1. Graphs that illustrate the principles used to identify the ideal mass of ^{57}Fe needed to provide a high percentage of the spectrum from the ^{57}Fe-labeled mineral while maintaining a minimal amount of sample in the Mössbauer sample holder for soils contaning (a) 0.5%, (b) 1.0%, (c) 3.8% and (d) 6 % Fe.

2.2 Calculation of reduction of added ferrihydrite

Considering that natural abundance Fe contains[7] 2.12% ^{57}Fe and that the labeled ferrihydrite contained 95.5% ^{57}Fe (measured by ICP-MS), we want to estimate the percentage of ^{57}Fe coming from the reduction of ferrihydrite (y) based on the percentage of ^{57}Fe contained in the aqueous phase (x). The percentage of ^{57}Fe in the aqueous phase can be calculated as follows:

$$x = \frac{\left(95.5y + 2.12(100 - y)\right)}{100}$$

Therefore knowing the percentage of ^{57}Fe in the aqueous phase (x), we can calculate the percentage of the aqueous phase derived from atoms initially in ferrihydrite:

$$y = 1.07x - 2.27$$

3 FERRIHYDRITE CHARACTERIZATION

Our ^{57}Fe-labeled laboratory synthesized ferrihydrite was confirmed to be a 2-line ferrihydrite by XRD, with no additional sharp peaks detected (**Figure S2**), which rules out the presence of other crystalline Fe phases.

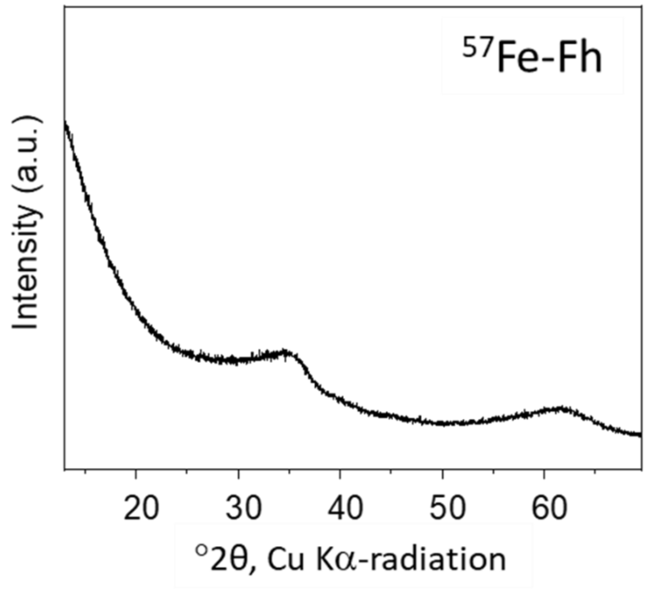

Figure S2. X-ray diffraction pattern of ^{57}Fe-labeled ferrihydrite.

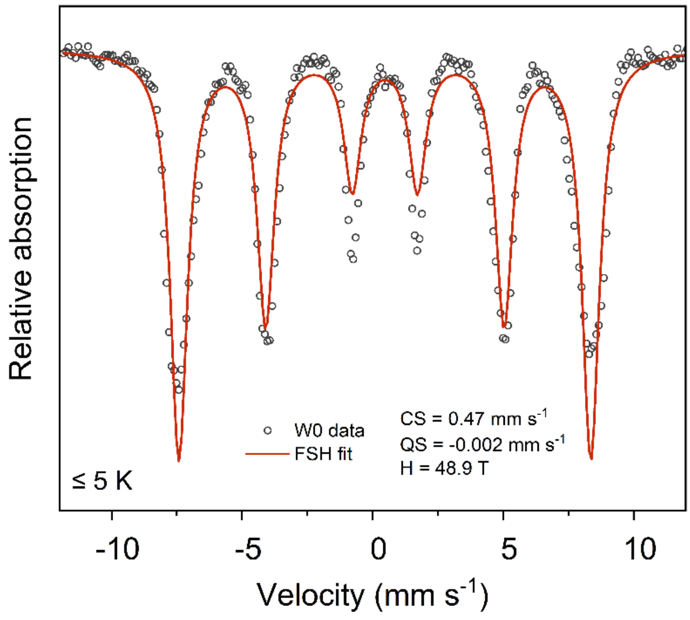

Figure S3. Fitted Mössbauer spectrum (≤ 5 K) of week 0 (initial) ^{57}Fe-ferrihydrite-soil mixture using Fully Static Hamiltonian (FSH) mode.

4 SOILS

This study included three soils and one sediment: a rice paddy soil used in the main experiment which is referred to as 'Paddy Soil', an intertidal flat sediment from Germany (referred to as ‘Intertidal Sediment’), a river floodplain soil from Switzerland (referred to as ‘Floodplain Soil’), and a clay-rich acid sulfate paddy soil from Thailand (referred to as ‘Acid Sulfate Soil’), All soils were dried at 30 °C, sieved through a 2 mm sieve, and stored in an ambient atmosphere until use.

4.1 Additional information and characterization of soils

Table S2. Information and characterization of soils used in this study.

		Soil			
		**Paddy Soil**	**Intertidal Sediment**	**Floodplain Soil**	**Acid Sulfate Soil**
**Location**		Ubon Ratchathani province, Thailand	Wadden sea (Friedrichskoog, Germany)	Thur Floodplain, Switzerland	Chachoengsao province, Thailand
**Soil classification (World Reference Base for Soil Resources)[8]**		Hydragric Siltic Anthrosol	Tidalic Gleysol	Haplic Fluvisol	Hydragric Vertic Anthrosol
**Coordinates**		15° 25’ 12” N<br>104° 34’ 48” E	54° 0’ 42” N<br>8° 50’ 6” E	47° 35’ 28” N<br>8° 46’ 27” E	14° 09’ 36” N<br>100° 48’ 36” E
**Sampling date**		Feb-2019	Aug-2020	Apr-2019	Feb-2019
**Soil Texture (Clay/Silt/Sand)**		Sandy loam (5/25/70) [c]	Silty/Sandy[d]	Silty[d]	Clay (61/36/2) [c]
**Soil pH[e]**		3.7	7.2	7.3	4.1
**N[a]**	**(%)**	0.06	0.04	0.09	0.28
**C [a]**	**(%)**	0.54	0.86	5.98	2.83
**Mg [b]**	**(%)**	<0.002	0.70	2.14	0.21
**Al [b]**	**(%)**	1.53	3.33	2.71	8.71
**Si [b]**	**(%)**	42.2	27.6	14.0	21.8
**P [b]**	**(%)**	0.03	0.06	0.08	0.04
**S [b]**	**(µg/g)**	324	4987	512	2625
**Ca [b]**	**(%)**	0.025	3.171	13.14	0.32
**Fe [b]**	**(%)**	0.55	0.92	1.15	3.80

[a] Measured with an organic elemental analyzer (vario MAX cube, Elementar, Germany)

[b] Measured with X-ray fluorescence spectrometry (XRF; XEPOS, Spectro) using pressed pellets of milled soil

[c] Soil texture measured by the Laboratoire et Bureau d’Etude au Service de l’Agriculture et de la Protection de l’Environnement (Gland, Switzerland)

[d] Texture based on field estimate

[e] The pH was measured with a 1:5 ratio of dry soil to 0.01 M $CaCl_2$

4.2 Mössbauer spectroscopy of soils

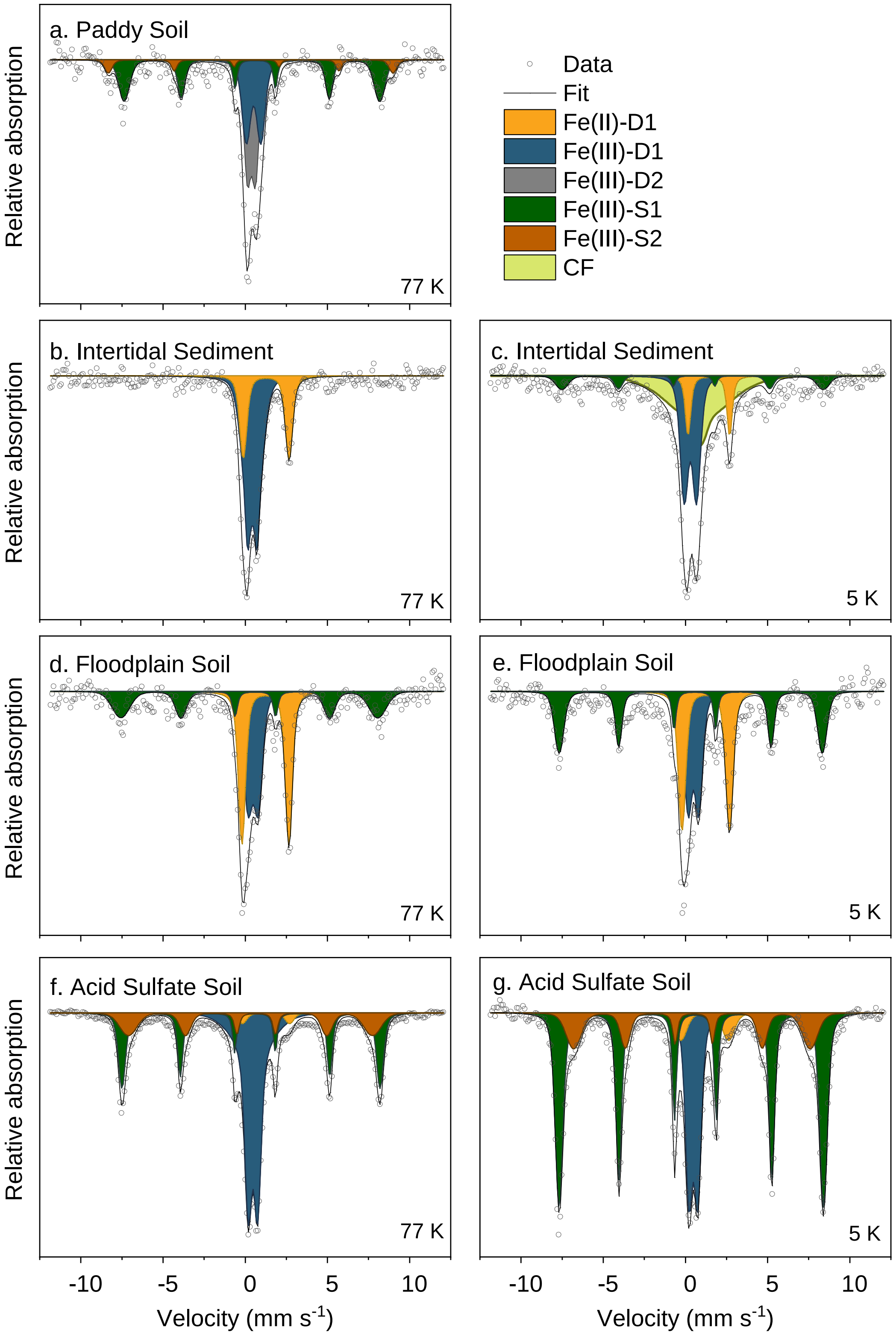

Figure S4. Mössbauer spectra and fits for initial (a) Paddy Soil, (b and c) Intertidal Sediment, (d and e) Floodplain Soil, and (f and g) Acid Sulfate Soil collected at 77 K and ≤ 5 K. Abbreviations: Fe(II)-D1 = Fe(II) doublet; Fe(III)-D1/2 = Fe(III) doublet; Fe(III)-S1/2 = Fe(III) sextet; CF = collapsed feature.

Table S3. Hyperfine parameters obtained for fitting of raw soils with xVBF model. Mössbauer spectra were collected at 77 K and ≤ 5 K.

Sample	Temp.	Phase	Phase interpretation	Spectral Area %	CS[a] (mm s^{-1})	QS or ε[b] (mm s^{-1})	H[c] (T)	σ[d] (mm s^{-1}) or (T)	Red-χ^2 [e]
**Paddy Soil**	77 K	Fe(III)-D1	Ferrihydrite or lepidocrocite	26.9	0.51	0.89	-	0.39	0.85
		Fe(III)-D2	Surface-complexed Fe(III) or Fe(III) in phyllosilicates	31.6	0.38	0.51	-	0.28	
		Fe(III)-S1	Goethite	33.7	0.51	-0.10	48.2	1.70	
		Fe(III)-S2	Hematite	7.8	0.51	-0.18	53.7	1.00	
**Intertidal Sediment**	77 K	Fe(III)-D1	-	68.9	0.44	0.75	-	0.50	0.59
		Fe(II)-D1	-	31.1	1.27	2.78	-	0.32	
	≤ 5 K	Fe(III)-D1	Surface-complexed Fe(III) or Fe(III) in phyllosilicates	31.7	0.30	0.75	-	0.35	0.67
		Fe(II)-D1	Fe(II) surface-complexed or in phyllosilicates	11.8	1.42	2.53	-	0.19	
		Fe(III)-S1	Goethite and or ferrihydrite	11.5	0.48	-0.05	49.3	2.00*	
		CF	Fe(II) and Fe(III) that accounts for Fe(II) octets and Fe(III) (oxyhydr)oxides near ordering temperature	45.1	0.78	0.00*	11.7	7.99	
**Floodplain Soil**	77 K	Fe(III)-D1	-	32.7	0.48	0.64	-	0.37	0.63
		Fe(II)-D1	-	38.6	1.23	2.86	-	0.30	
		Fe(III)-S1	-	28.7	0.43	-0.18	48.4	2.97	
	≤ 5 K	Fe(III)-D1	Surface-complexed Fe(III) or Fe(III) in phyllosilicates(possibly some pyrite)	29.2	0.49	0.65	-	0.33	1.15
		Fe(II)-D1	Fe(II) surface-complexed or in phyllosilicates	34.3	1.24	2.89	-	0.33	
		Fe(III)-S1	Goethite and or ferrihydrite	36.5	0.45	-0.13	49.6	1.39	
**Acid Sulfate Soil**	77 K	Fe(III)-D1	-	47.7	0.47	0.99	-	1.02	1.82
		Fe(II)-D1	-	2.5	1.26	2.86	-	0.40*	
		Fe(III)-S1	-	30.4	0.47	-0.12	48.7	0.95	
		Fe(III)-S2	-	19.4	0.49	-0.17	46.1	2.93	
	≤ 5 K	Fe(III)-D1	Surface-complexed Fe(III) or Fe(III) in phyllosilicates(possibly some pyrite)	23.0	0.49	0.55	-	0.28	2.45
		Fe(II)-D1	Fe(II) surface-complexed or in phyllosilicates	5.3	1.17	2.90	-	0.60	
		Fe(III)-S1	Goethite	54.1	0.48	-0.12	49.9	1.08	
		Fe(III)-S2	Mixture of ferrihydrite, nanogoethite, lepidocrocite and possible iron sulfate minerals	17.5	0.45	-0.06	44.6	2.50	

* Indicates values that were fixed during the fitting process.
[a]Center shift;
[b]Quadrupole splitting (QS, for doublets) or quadrupole shift (ε, for sextets);
[c]Hyperfine field;
[d]σ, standard deviation of QS (doublet) or H (sextet);
[e]Red- χ^2, goodness of fit;
Abbreviations: CF = collapsed feature; Temp. = temperature; Fe(III)-D1/2 = Fe(III) doublet; Fe(III)-S1/2 = Fe(III) sextet; Fe(II)-D1 = Fe(II) doublet.

4.2.1 Paddy Soil

The 77 K Mössbauer spectrum of the Paddy Soil prior to ferrihydrite enrichment (**Figure S4a**) consisted of two sextets (Fe(III)-S1 and Fe(III)-S2)) and two Fe(III) doublets (Fe(III)-D1 and Fe(III)-D2)). The sextets present fitting parameters likely compatible with goethite (Fe(III)-S1), and hematite of medium crystallinity (Fe(III)-S2).[9] The Fe(III)-D1 is a paramagnetic Fe(III) at 77 K, which can indicate ferrihydrite or lepidocrocite,[10] and the Fe(III)-D2 has fitting parameters consistent with surface-complexed Fe(III) or Fe(III) in phyllosilicates.[11, 12] While analyzing Mössbauer spectra at additional temperatures would be beneficial for a more decisive identification of the Fe phases, the low Fe content in the samples prevented us from collecting additional spectra.

4.2.2 Intertidal Sediment

The 77 K Mössbauer spectrum of the Intertidal Sediment prior to ferrihydrite enrichment (**Figure S4b**) consisted of an Fe(II) and an Fe(III) paramagnetic doublet (Fe(II)-D1 and Fe(III)-D1). An additional spectrum at ≤ 5 K (**Figure S4c**) revealed an Fe(II) doublet and an Fe(III) doublet with fitting parameters compatible with Fe(II) and Fe(III) in phyllosilicates[11] (Fe(II)-D1 and Fe(III)-D1). Additionally, at ≤ 5 K, there is a small Fe(III) sextet (Fe(III)-S1), compatible with a mixture of goethite and ferrihydrite,[10, 13] a collapsed phase, likely a mix of Fe(II) and Fe(III) that accounts for Fe(II) octets and Fe(III) (oxyhydr)oxides near ordering temperature.

4.2.3 Floodplain Soil

The 77 K Mössbauer spectrum of the Floodplain Soil prior to ferrihydrite enrichment (**Figure S4d**) consisted of an Fe(II) and an Fe(III) paramagnetic doublets (Fe(II)-D1 and Fe(III)-D1), along with an Fe(III) sextet (Fe(III)-S1). An additional spectrum at ≤ 5 K (**Figure S4e**) revealed an Fe(II)-D1 and an Fe(III)-D1 with fitting parameters compatible with Fe(II) and Fe(III) in phyllosilicates.[11] Additionally, at ≤ 5 K, there is an Fe(III) sextet (Fe(III)-S1), compatible with a mixture of goethite and ferrihydrite.[10]

4.2.4 Acid Sulfate Soil

The 77 K Mössbauer spectrum of the Acid Sulfate Soil prior to ferrihydrite enrichment (**Figure S4f**) consisted of an Fe(II) and an Fe(III) paramagnetic doublet (Fe(II)-D1 and Fe(III)-D1), along with two Fe(III) sextets (Fe(III)-S1 and Fe(III)-S2). An additional spectrum at ≤ 5 K (**Figure S4g**) revealed an Fe(II) and an Fe(III) doublet with fitting parameters compatible

with Fe(II) and Fe(III) in phyllosilicates[11] (Fe(II)-D1 and Fe(III)-D1). Additionally, at ≤ 5 K, there is an Fe(III) sextet (Fe(III)-S1) with fitting parameters compatible with goethite and a second one (Fe(III)-S2) compatible with a mixture of ferrihydrite, nanogoethite, and lepidocrocite.[10] Since goethite, schwertmannite, and jarosite have 5 K Mössbauer parameters that can overlap with each other, we cannot exclude the possibility that a fraction of the sextet comprises Fe (hydroxy)sulfate minerals.[14]

5 ROBUSTNESS OF THE MICROCOSM EXPERIMENT

In the process of optimizing the microcosm experiment, we tested multiple conditions, such as the use of 0.5 mM $CaCl_2$ versus UPW, parafilm sealed versus capped tubes, and agitation versus non-agitation of the samples. For this purpose, we prepared two reactors (one for each time point) containing 0.5 mM $CaCl_2$, closed with parafilm, and not agitated. Additionally, each of the conditions were tested in isolation. To test for the influence of $CaCl_2$, we additionally prepared three samples using 15 mL anoxic UPW instead of 0.5 mM $CaCl_2$ (parafilm and non-agitated). To test for the sealing method, three samples were prepared with tightly closed caps instead of using parafilm (incubated with 0.5 mM $CaCl_2$, non-agitated). To test for agitation, in addition to having the caps closed, three samples were placed horizontally on an orbital shaker at 200 rpm for the duration of the experiment (0.5 mM $CaCl_2$). A summary of experimental conditions is provided in **Table S3**. Sacrificial reactors were sampled at 4 (one sample) and 12 (two samples, one for samples with 0.5 mM $CaCl_2$, closed with parafilm, and not agitated) weeks using the sampling procedure described in the main text.

Comparison between aqueous phase characterization of incubations using 0.5 mM $CaCl_2$, parafilm, and non-agitation of the samples and incubations using UPW (instead of $CaCl_2$), closed caps (instead of parafilm), and agitated samples (instead of non-agitated), respectively, revealed no substantial changes to the aqueous phase (**Figures S6 and S7).** The 77 K Mössbauer spectra of all samples used in the comparisons at 4 weeks and 12 weeks are shown in **Figures S5a and b**, and additional measurements at $\leq$ 5 K are shown in **Figures S5c and d**. The collected data of samples using UPW, closed caps, and agitated samples were overlaid with the samples using 0.5 mM $CaCl_2$, parafilm, and non-agitation of the samples in the same weeks for direct comparison.

The first condition tested was the use of 0.5 mM $CaCl_2$, which was used to provide a background electrolyte to keep soil colloids aggregated. While such addition is beneficial to preserving soil properties, the excess of anions could affect the transformation of Fe oxides.[15] The aqueous phase of soils incubated with UPW water was more turbid than the aqueous phase using $CaCl_2$ (**Figures S17**). Hence, $CaCl_2$ helped prevent the dispersion of colloids. However, the Cl^- concentration in the aqueous phase was in the same order of magnitude in samples incubated with UPW, compared to $CaCl_2$, suggesting most of the Cl^- came from the soil and not from the initial composition of the aqueous phase. In experiments with UPW, Ca^+ concentrations were half the concentrations of reactors incubated with 0.5 mM $CaCl_2$ (**Figure S7**). Nonetheless, the Mössbauer spectra of samples incubated with UPW or 0.5 mM $CaCl_2$ are

nearly identical at 4 or 12 weeks (**Figure S5**). Therefore, we concluded that the use of 0.5 mM $CaCl_2$ did not affect the transformation of ferrihydrite in our experimental conditions.

The second experimental condition tested was the use of parafilm, which has permeability to CO_2 (400 cc/m^2/24 h at 22.8 °C, 50% relative humidity, corrected to 760 mm pressure, Parafilm® M – product information sheet). The use of parafilm theoretically allows for CO_2 escape, likely preventing the preferential formation of siderite due to CO_2 buildup.[16] To assess whether the use of parafilm affected ferrihydrite transformation, we compared incubations using parafilm with incubations with closed caps. We found no substantial changes in aqueous phase characterization (**Figures S6 and S7**). However, we did not measure dissolved inorganic carbon or alkalinity in our experiment. Nonetheless, Mössbauer results demonstrated that allowing for CO_2 escape did not change ferrihydrite transformation products or transformation extent at 4 or 12 weeks (**Figure S5**).

Finally, the third experimental condition tested was the agitation of samples. Since agitation can influence the rate of microbial activity,[17, 18] we tested the effect of shaking samples on the transformation of ferrihydrite. Despite leading to more suspended solids in the aqueous phase (**Figure S17**), we found no evidence of changes in the composition of the aqueous phase or in ferrihydrite transformation products or transformation extent at 4 or 12 weeks (**Figures S5, S6, and S7**).

In summary, the tests conducted during method development demonstrate that different experimental conditions may be suitable for our proposed methodology in microcosms. This attests to the flexibility and robustness of the method. For this study, we decided to use the experimental conditions of 0.5 mM $CaCl_2$, parafilm, and not agitating the incubations.

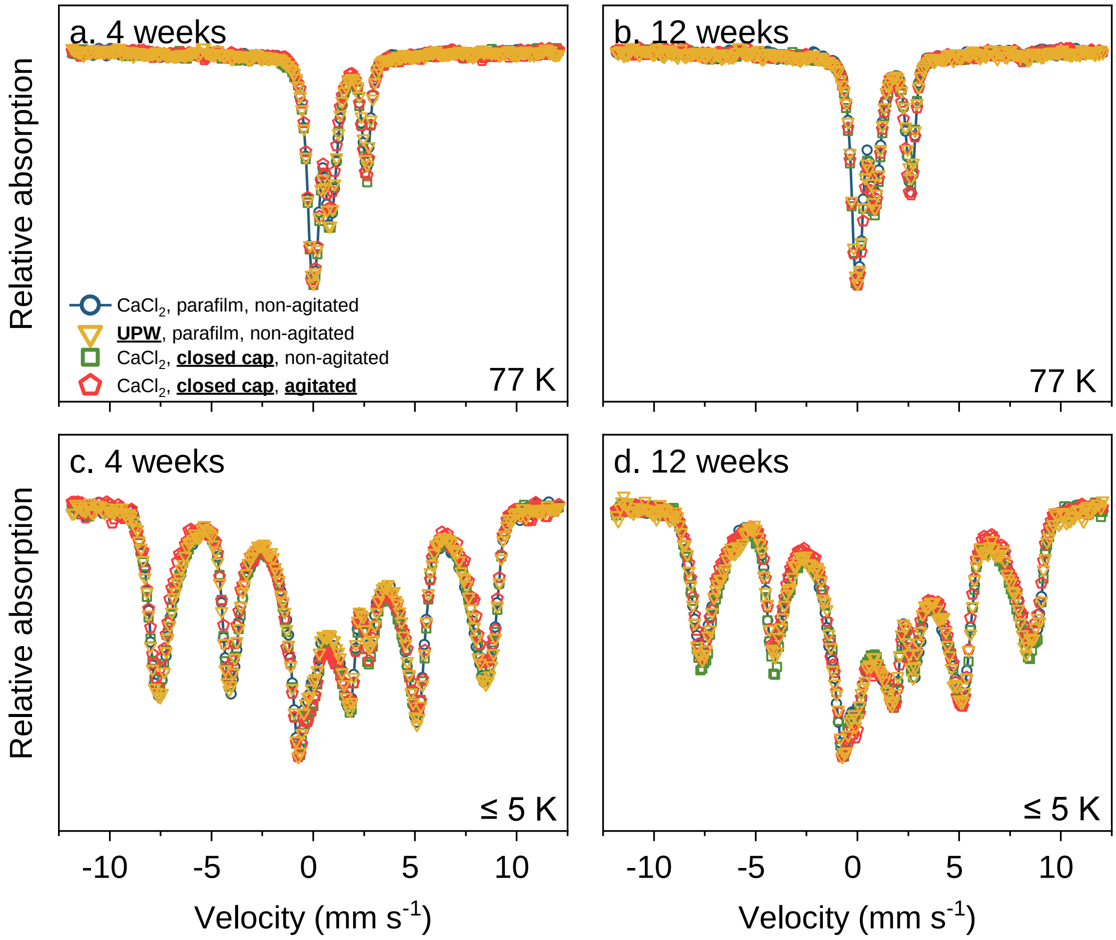

Figure S5. Comparison of Mössbauer spectra collected for paddy soil spiked with ^{57}Fe-ferrihydrite incubated using varying conditions. Samples were taken at 4 and 12 weeks, and spectra were collected at 77 and ≤ 5 K. Spectra are overlaid for comparison, and the experimental condition underlined highlights the tested condition.

6 AQUEOUS PHASE ANALYSIS

6.1 Aqueous Fe(II) to Fe total (FeT)ratio

Table S4. $Fe(II)_{aq}$:$Fe(T)_{aq}$ ratio calculated based on results from aqueous Fe(II) and FeT concentration using the 1,10-phenanthroline protocol.[1]

Soil (experimental details)	Incubation time	Aqueous		
		Fe(II)	Fe(total)	$Fe(II)_{aq}$:$Fe(T)_{aq}$
	(weeks)	uM Fe	uM Fe	(%)
**Paddy Soil (Main experiment)**	0	0	0	-
**Paddy Soil (Main experiment)**	1	1718	1741	99
**Paddy Soil (Main experiment)**	2	2051	1923	107
**Paddy Soil (Main experiment)**	4	1070	1046	102
**Paddy Soil (Main experiment)**	8	678	739	92
**Paddy Soil (Main experiment)**	12	677	629	108
**Paddy Soil (Main experiment)**	16	726	700	104
**Paddy Soil (Main experiment)**	16	763	758	101
**Paddy Soil (UPW)**	4	488	487	100
**Paddy Soil (UPW)**	12	281	265	106
**Paddy Soil (UPW)**	12	450	445	101
**Paddy Soil (closed cap)**	4	776	765	101
**Paddy Soil (closed cap)**	12	318	296	108
**Paddy Soil (closed cap)**	12	499	503	99
**Paddy Soil (agitated)**	4	1035	1036	100
**Paddy Soil (agitated)**	12	835	848	99
**Paddy Soil (agitated)**	12	787	794	99
**Paddy Soil (No Fh control)**	6	746	714	104
**Paddy Soil (No Fh control)**	12	435	387	112
**Paddy Soil (No Fh control)**	12	439	425	103
**Intertidal Sediment - ^{57}Fe-ferrihydrite**	4	157	137	115
**Intertidal Sediment - ^{NA}Fe-ferrihydrite**	4	**	**	**
**Intertidal Sediment - ^{57}Fe-ferrihydrite**	12	39*	3*	-
**Intertidal Sediment - ^{NA}Fe-ferrihydrite**	12	46*	17*	-
**Floodplain Soil - ^{57}Fe-ferrihydrite**	4	82*	51*	-
**Floodplain Soil - ^{NA}Fe-ferrihydrite**	4	139*	105*	-
**Floodplain Soil - ^{57}Fe-ferrihydrite**	12	1989	1974	101
**Floodplain Soil - ^{NA}Fe-ferrihydrite**	12	618	583	106
**Acid Sulfate Soil - ^{57}Fe-ferrihydrite**	4	5170	5295	98
**Acid Sulfate Soil - ^{NA}Fe-ferrihydrite**	4	**	**	**
**Acid Sulfate Soil - ^{57}Fe-ferrihydrite**	12	71*	32*	-
**Acid Sulfate Soil - ^{NA}Fe-ferrihydrite**	12	61*	23*	-

* Analysis used 20x dilution. Therefore, values smaller than 140 uM Fe led to significant errors. The $Fe(II)_{aq}$:$Fe(T)_{aq}$ ratio for these samples were disregarded.
**Missing values

Table S5. Fe and ^{57}Fe mass balance in aqueous and solids phase.

Soil (experimental details)	Incub. time	Solids				Aqueous phase				Cal. Fe in solid phase		
		Fe in soil	Fe in added Fh[a]	^{57}Fe in soil[b]	^{57}Fe in Fh	Fe_{aq}[c]	Fe in aqueous phase[d] (in 15 mL)	$^{57}Fe/Fe_T$ in aqueous phase[e]	^{57}Fe in aqueous phase (in 15 mL)	Fe left in solid phase	^{57}Fe left in solid phase	
	(weeks)	(mg)	(mg)	(mg)	(mg)	uM	(mg)	%	(mg)	(mg)	(mg)	(%)
**Paddy Soil (ME)**	0	82.5	43.3	1.8	41.1	0	0.0	0.025	0.0	125.8	42.9	100.0
**Paddy Soil (ME)**	1	x	x	x	x	2044	1.7	0.645	1.1	124.1	41.8	97.4
**Paddy Soil (ME)**	2	x	x	x	x	2266	1.9	0.713	1.4	123.9	41.5	96.8
**Paddy Soil (ME)**	4	x	x	x	x	1208	1.0	0.691	0.7	124.8	42.2	98.4
**Paddy Soil (ME)**	8	x	x	x	x	860	0.7	0.670	0.5	125.1	42.4	98.9
**Paddy Soil (ME)**	12	x	x	x	x	756	0.6	0.668	0.4	125.2	42.5	99.0
**Paddy Soil (ME)**	16	x	x	x	x	805	0.7	0.670	0.5	125.1	42.4	98.9
**Paddy Soil (ME)**	16	x	x	x	x	841	0.7	0.641	0.5	125.1	42.4	98.9
**Paddy Soil (UPW)**	4	x	x	x	x	608	0.5	0.683	0.3	125.3	42.5	99.2
**Paddy Soil (UPW)**	12	x	x	x	x	361	0.3	0.657	0.2	125.5	42.7	99.5
**Paddy Soil (UPW)**	12	x	x	x	x	579	0.5	0.649	0.3	125.3	42.6	99.3
**Paddy Soil (closed cap)**	4	x	x	x	x	963	0.8	0.650	0.5	125.0	42.3	98.8
**Paddy Soil (closed cap)**	12	x	x	x	x	406	0.3	0.662	0.2	125.4	42.6	99.5
**Paddy Soil (closed cap)**	12	x	x	x	x	622	0.5	0.672	0.4	125.3	42.5	99.2
**Paddy Soil (agitated)**	4	x	x	x	x	1259	1.1	0.698	0.7	124.7	42.1	98.3
**Paddy Soil (agitated)**	12	x	x	x	x	1053	0.9	0.682	0.6	124.9	42.3	98.6
**Paddy Soil (agitated)**	12	x	x	x	x	995	0.8	0.678	0.6	125.0	42.3	98.7
**Intertidal flat sed. - ^{57}Fe-ferrihydrite**	0	138.0	43.3	3.0	41.1	0	0.0	0.025	0.0	181.3	44.1	100.0
**Intertidal flat sed. - ^{57}Fe-ferrihydrite**	4	x	x	x	x	188	0.2	0.667	0.1	181.1	43.9	99.8
**Intertidal flat sed. - ^{57}Fe-ferrihydrite**	12	x	x	x	x	43	0.0	0.669	0.0	181.3	44.0	99.9
**Floodplain Soil - ^{57}Fe-ferrihydrite**	0	172.5	43.3	3.8	41.1	0	0.0	0.025	0.0	215.8	44.8	100.0
**Floodplain Soil - ^{57}Fe-ferrihydrite**	4	x	x	x	x	116	0.1	0.687	0.1	215.7	44.7	99.9
**Floodplain Soil - ^{57}Fe-ferrihydrite**	12	x	x	x	x	83	0.1	0.436	0.0	215.7	44.8	99.9
**Acid Sulfate Soil - ^{57}Fe-ferrihydrite**	0	570.0	76.6	12.5	72.8	0	0.0	0.025	0.0	646.6	84.8	100.0
**Acid Sulfate Soil - ^{57}Fe-ferrihydrite**	4	x	x	x	x	6850	5.8	0.326	1.9	640.8	83.0	97.8
**Acid Sulfate Soil - ^{57}Fe-ferrihydrite**	12	x	x	x	x	561	0.5	0.272	0.1	646.1	84.7	99.8

[a]Considering Fe to account for 66% of the mass of Fh;
[b]Considering ^{NA}Fe to have 2.12% ^{57}Fe;
[c]Assuming there was 15 mL of aqueous phase at all sampling times;
[d]From ICP-OES analysis;
[e]From ICP-MS analysis;
Abbreviations: Incub. = Incubation; Sed. = sediment; Cal. = calculations; ME = main experiment.
Values not measured or that could not be calculated are marked with an 'x'

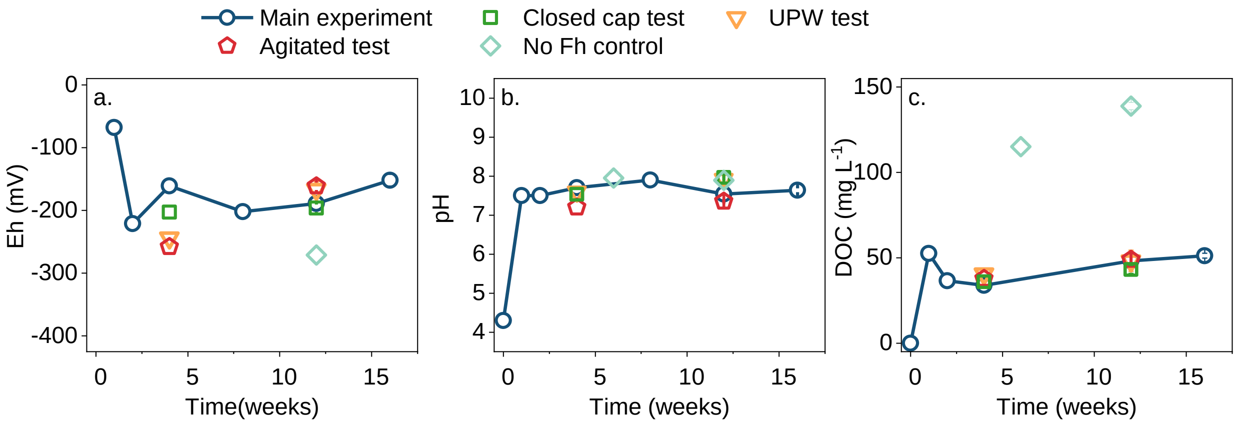

Figure S6. Aqueous phase values of (a) Eh, (b) pH, and (c) dissolved organic carbon concentration (DOC) during 16 weeks of incubation of Paddy Soil spiked with ^{57}Fe-ferrihydrite in the main experiment and robustness tests. Notes: Eh value was not measured at day zero and the pH value for day zero was measured 3 hours after incubation. For samples of 16-week (main experiment) and 12-week (no Fh control, UPW, closed cap, and agitation tests), the marker represents the average of duplicates, and the error bars represent the standard deviation. Eh and pH data for main experiment is also shown in **Figure 1**.

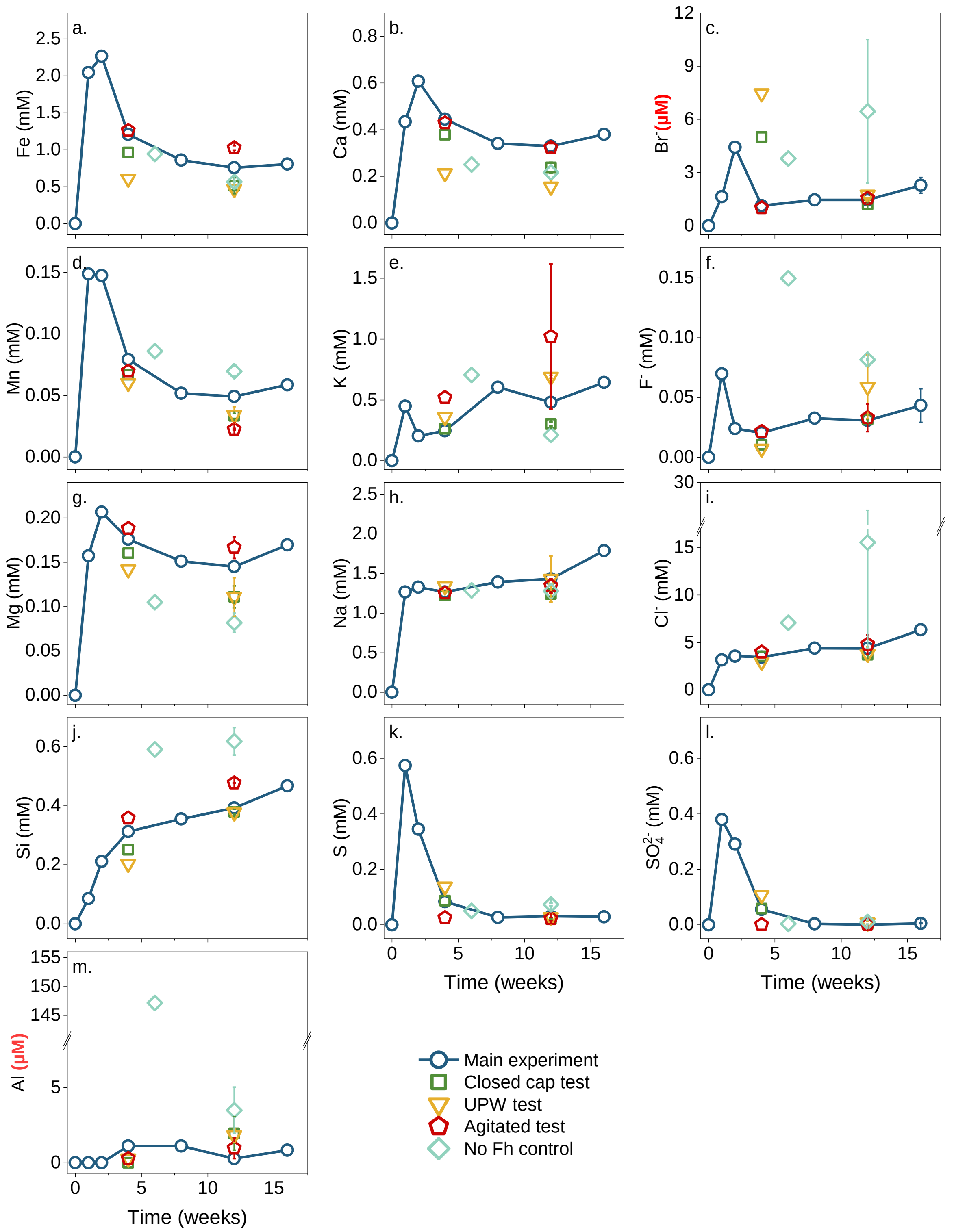

Figure S7. Major anions and cations in the aqueous phase of incubation with Paddy Soil spiked with ^{57}Fe-ferrihydrite in the main experiment and robustness tests. Note that the y-axis marked in red are in µM. For samples of 16-week (main experiment) and 12-week (no Fh control, UPW, closed cap, and agitation tests), the marker represents the average of duplicates, and the error bars represent the standard deviation.

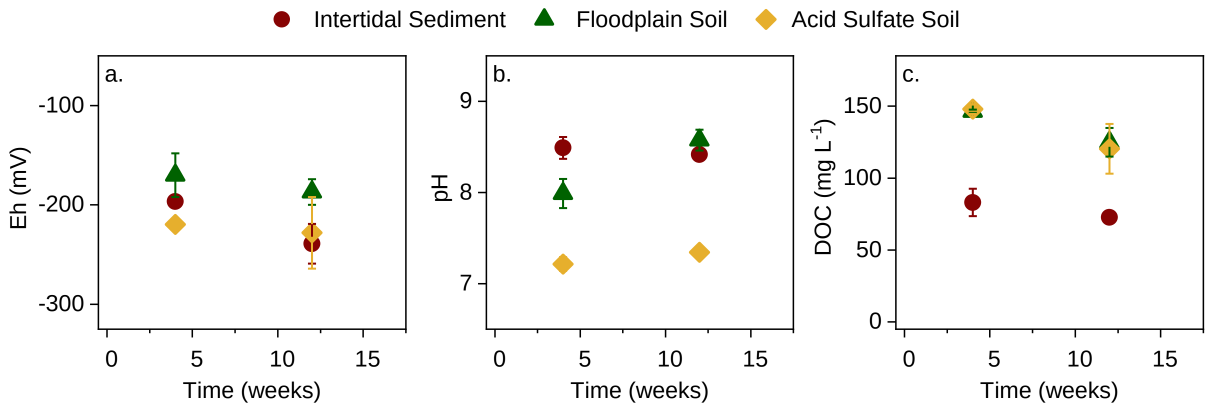

Figure S8. Aqueous phase values of (a) Eh, (b) pH, and (c) DOC during 12 weeks of incubation of Intertidal Sediment, Floodplain Soil, and Acid Sulfate Soil spiked with ^{57}Fe-ferrihydrite.

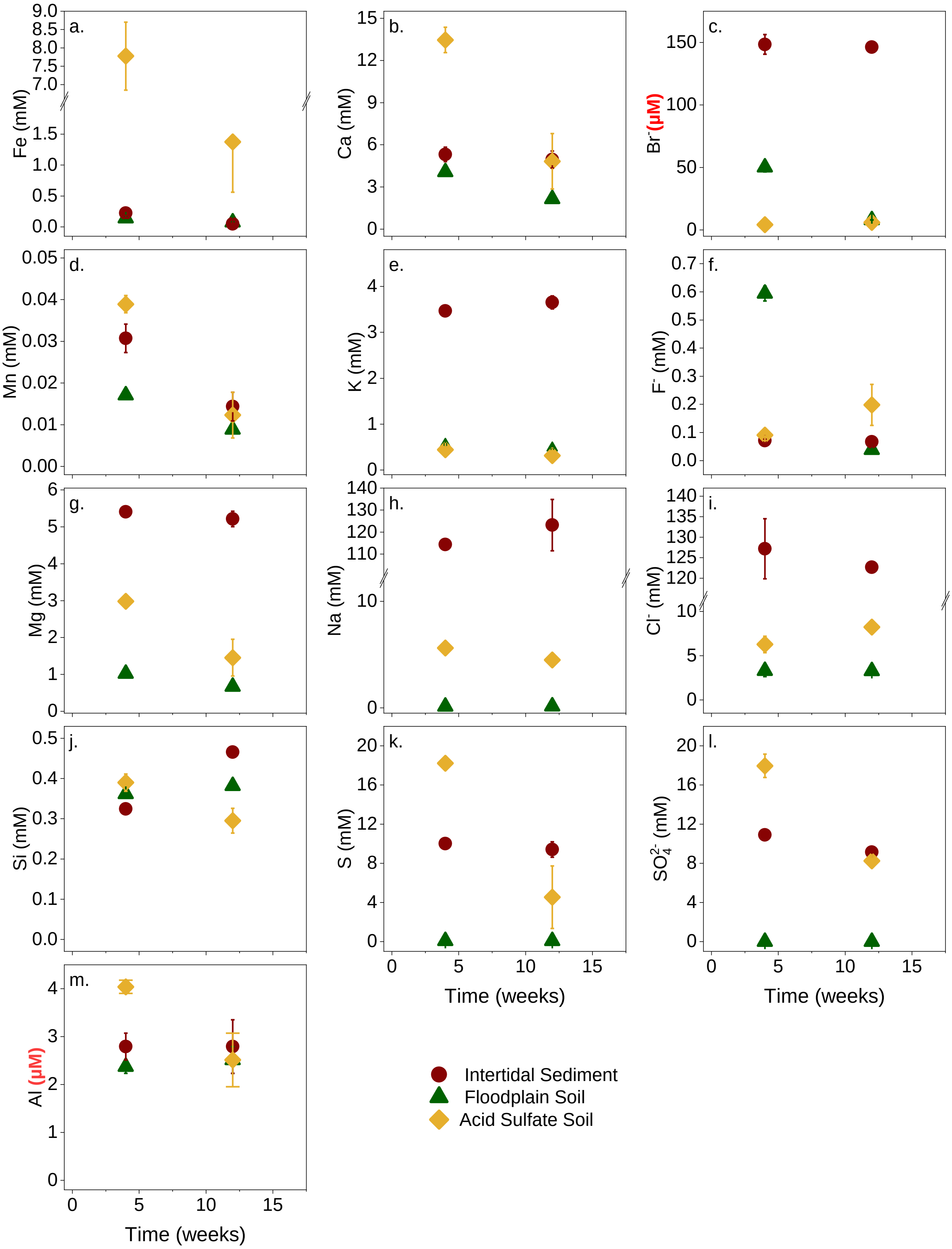

Figure S9. Major anions and cations in the aqueous phase of incubation with Intertidal Sediment, Floodplain Soil, and Acid Sulfate Soil spiked with ^{57}Fe-ferrihydrite. Note that the y-axis marked in red is in µM. The markers represent the average of duplicates, and the error bars represent the standard deviation. Error bars not seen are smaller than the marker.

7 MÖSSBAUER SPECTROSCOPY

7.1 Paddy Soil incubation

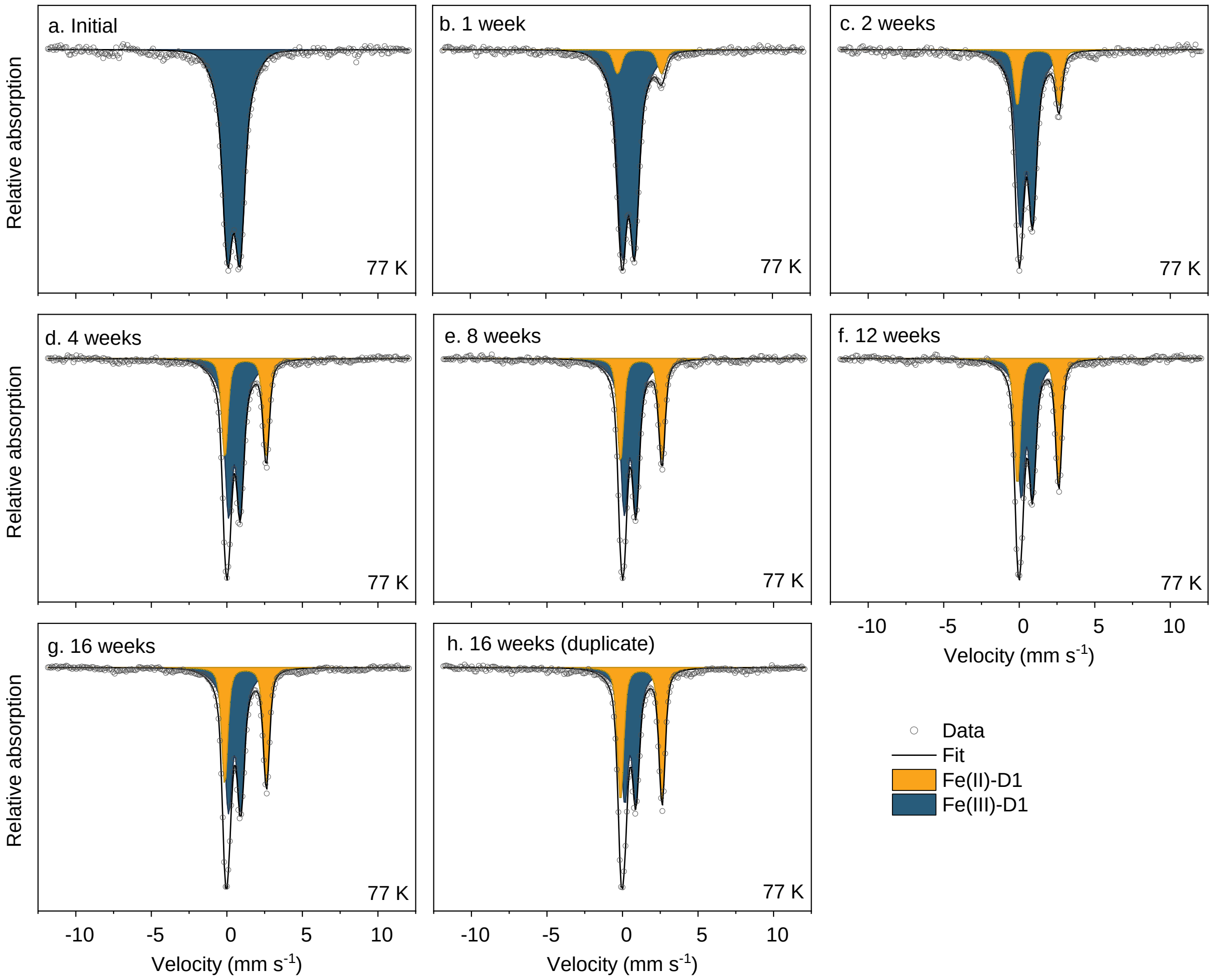

Figure S10. Mössbauer spectra of Paddy Soil spiked with ^{57}Fe-ferrihydrite during 16-week incubation. Abbreviations: Fe(II)-D = Fe(II) doublet; Fe(III)-D = Fe(III) doublet. Panels a, b, d, and g are also shown in **Figure 2**.

Table S6. Hyperfine parameters obtained for fitting (xVBF model) of Paddy Soil spiked with ^{57}Fe-ferrihydrite during 16-week incubation. Spectra collected at 77 K.

Sample	Phase	Spectral Area %	CS[a] (mm s^{-1})	QS[b] (mm s^{-1})	σ[c] (mm s^{-1}) or (T)	Red- χ^2 [e]
W0	Fe(III)-D1	100.0	0.45	1.12	0.85	0.66
	comp. 1	*66.0*	*0.45*	*0.83*	*0.50*	
	comp. 2	*34.0*	*0.45*	*1.00**	*2.00**	
W1	Fe(III)-D1	91.3	0.47	1.17	10.98	0.78
	comp. 1	*64.4*	*0.47*	*0.81*	*0.44*	
	comp. 2	*35.8*	*0.47*	*1.00**	*2.00**	
	Fe(II)-D1	8.7	1.21	2.92	0.41	
W2	Fe(III)-D1	80.8	0.47	1.13	0.93	0.76
	comp. 1	*68.7*	*0.47*	*0.82*	*0.39*	
	comp. 2	*31.3*	*0.47*	*1.00**	*2.00**	
	Fe(II)-D1	19.2	1.25	2.76	0.30	
W4	Fe(III)-D1	67.5	0.50	1.09	0.93	1.71
	comp. 1	*68.8*	*0.50*	*0.77*	*0.34*	
	comp. 2	*31.2*	*0.50*	*1.00**	*2.00**	
	Fe(II)-D1	32.5	1.24	2.74	0.28	
W8	Fe(III)-D1	64.9	0.50	1.03	0.85	1.39
	comp. 1	*74.9*	*0.50*	*0.77*	*0.34*	
	comp. 2	*25.1*	*0.50*	*1.00**	*2.00**	
	Fe(II)-D1	35.2	1.24	2.75	0.28	
W12	Fe(III)-D1	57.1	0.51	1.00	0.84	1.32
	comp. 1	*76.1*	*0.51*	*0.75*	*0.32*	
	comp. 2	*23.9*	*0.51*	*1.00**	*2.00**	
	Fe(II)-D1	42.9	1.25	2.75	0.27	
W16'	Fe(III)-D1	61.5	0.51	1.08	0.86	3.19
	comp. 1	*73.4*	*0.51*	*0.81*	*0.34*	
	comp. 2	*26.6*	*0.51*	*1.00**	*2.00**	
	Fe(II)-D1	38.5	1.24	2.77	0.27	
W16''	Fe(III)-D1	54.8	0.51	0.98	0.82	1.2
	comp. 1	*77.4*	*0.51*	*0.74*	*0.33*	
	comp. 2	*22.6*	*0.51*	*1.00**	*2.00**	
	Fe(II)-D1	45.3	1.25	2.75	0.28	

[a]Center shift;
[b]Quadrupole splitting;
[c]σ, standard deviation of QS;
[e]Red- χ^2, goodness of fit;
* Indicates values that were fixed during the fitting process.
Abbreviations: Comp. = component; Fe(II)-D = Fe(II) doublet; Fe(III)-D = Fe(III) doublet; WX = week x.
For some broad doublets, we deployed a two-component fit to describe the population distribution. Parameters marked in red are the components of the previous phase. Note that the percentage of components always sums to 100% but refers to the percentage of the previous phase.

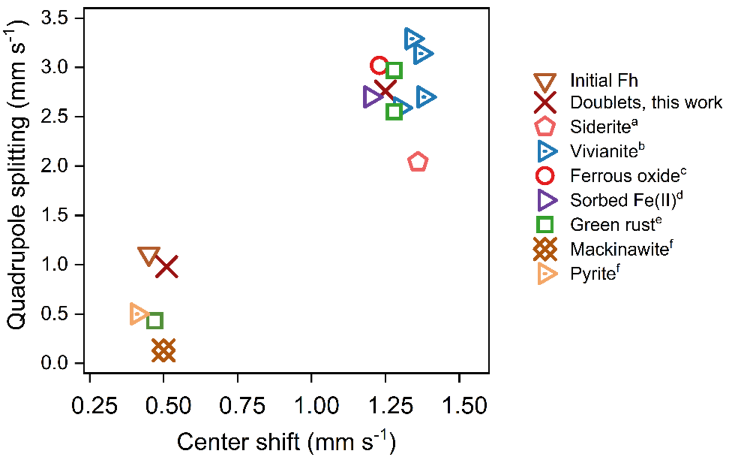

Figure S11. Summary of Mössbauer fitting parameters (77 K) of initial ferrihydrite, doublets at 16 weeks, and common Fe species reported in the literature. References a = [9], b = [19], c = [20], d =[21], e = [22], f =[23].

7.2 Paddy Soil incubation – temperature profile

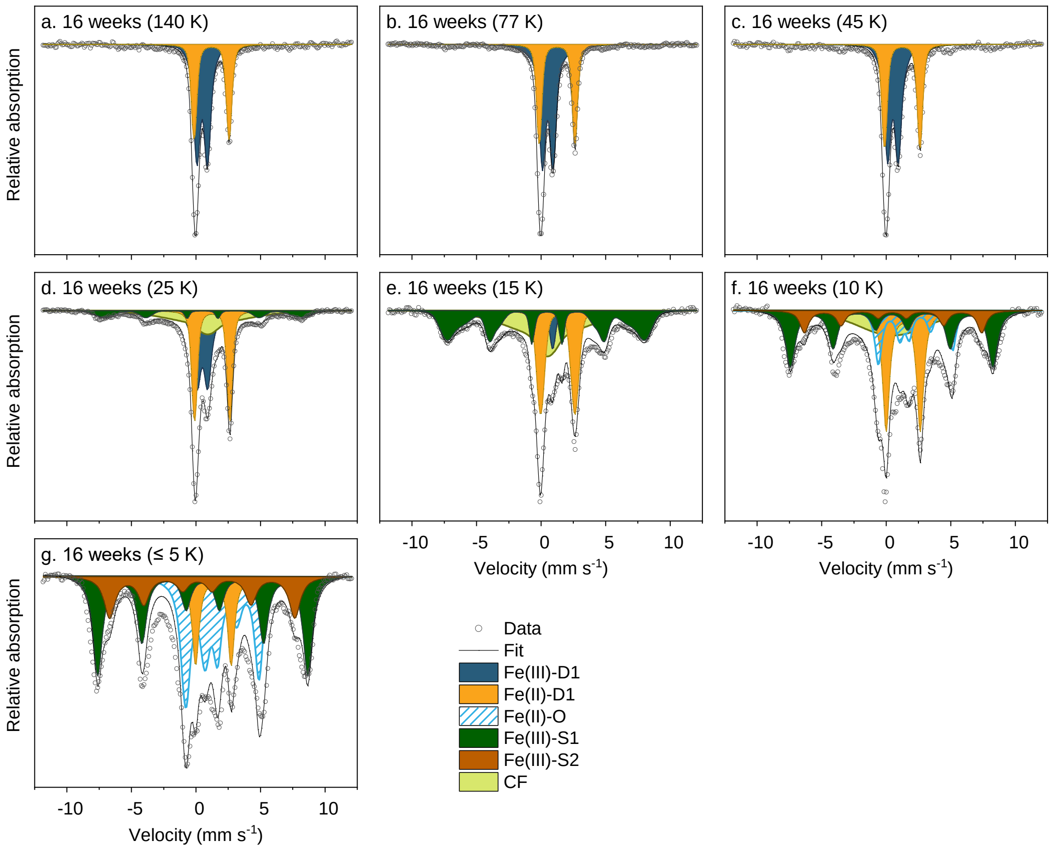

Figure S12. Mössbauer spectra of the temperature profile of Paddy Soil spiked with ^{57}Fe-ferrihydrite incubated for 16 weeks. Abbreviations: Fe(II)-D1 = Fe(II) doublet; Fe(III)-D1/2 = Fe(III) doublet; Fe(III)-S1/2 = Fe(III) sextet; CF = collapsed feature. Panel b is also shown in **Figure S10**, and panels d, f, and g are also shown in **Figure 2**.

Table S7. Hyperfine parameters obtained for fitting (xVBF model) of spectra of Paddy Soil spiked with ^{57}Fe-ferrihydrite for 16 weeks. Spectra collected at 140, 77, 45, 25, and 15 K.

Temp.	Phase	Spectral Area %	CS[a] (mm s^{-1})	QS or ε[b] (mm s^{-1})	σ[d] (mm s^{-1}) or (T)	H[c] (T)	Red- χ^2 [e]
140 K	Fe(III)-D1	61.6	0.48	1.04	0.81	x	1.03
	comp. 1	*78.0*	*0.48*	*0.82*	*0.37*	*x*	
	comp. 2	*21.9*	*0.48*	*1.00**	*2.00**	*x*	
	Fe(II)-D2	38.4	1.21	2.72	0.30	*x*	
77 K	Fe(III)-D1	61.5	0.51	1.08	0.86	*x*	3.19
	comp. 1	*73.4*	*0.51*	*0.81*	*0.34*	*x*	
	comp. 2	*26.6*	*0.51*	*1.00**	*2.00**	*x*	
	Fe(II)-D1	38.5	1.24	2.77	0.27	*x*	
45 K	Fe(III)-D1	60.4	0.52	1.11	0.90	x	1.3
	comp. 1	*70.1*	*0.52*	*0.82*	*0.35*	*x*	
	comp. 2	*29.9*	*0.52*	*1.00**	*2.00**	*x*	
	Fe(II)-D2	39.6	1.24	2.75	0.28	x	
25 K	Fe(III)-D1	31.6	0.51	1.19	1.00	x	1.96
	comp. 1	*61.5*	*0.51*	*0.82*	*0.39*	*x*	
	comp. 2	*38.5*	*0.51*	*1.00**	*2.00**	*x*	
	Fe(II)-D2	30.8	1.26	2.74	0.30	x	
	Fe(III)-S1	11.1	0.53	0.00*	46.85	4.2	
	CF	26.6	0.84	0.00*	19.72	14.4	
15 K	Fe(III)-D1	6.7	0.41	0.985	0.29	x	3.76
	Fe(II)-D2	25.6	1.29	2.657	0.50	x	
	Fe(III)-S1	33.8	0.41	-0.041	5.05	44.9	
	comp. 1	*63.5*	*0.41*	*-0.041*	*3.00**	*47.8*	
	comp. 2	*36.5*	*0.41*	*-0.041*	*4.00**	*40.0**	
	CF	33.9	0.50*	0.00*	20.00*	15.0*	

[a]Center shift;
[b]Quadrupole splitting (QS, for doublets) or quadrupole shift (ε, for sextets);
[c]Hyperfine field;
[d]σ, standard deviation of QS (doublets) or H (sextets);
[e]Red- χ^2, goodness of fit;
Abbreviations: Temp. = temperature; Comp. = component; Fe(II)-D = Fe(II) doublet; Fe(III)-D = Fe(III) doublet; Fe(III)-S = Fe(II) sextet, CF = collapsed feature.
* Indicates values that were fixed during the fitting process.
For some broad doublets or sextets, we deployed a two-component fit to describe the population distribution. Parameters marked in red are the components of the previous phase. Note that the percentage of components always sums to 100% but refers to the percentage of the previous phase.

Table S8. Hyperfine parameters obtained for fitting (Full Static Hamiltonian model) of Paddy Soil spiked with ^{57}Fe-ferrihydrite for 16 weeks. Spectra collected at 10 and ≤ 5K.

Temp.	Phase	Phase interpretation	Spectral Area %	CS[a] (mm s^{-1})	H[b] (T)	$e^2qQ/2$[c] (mm s^{-1})	η[d] (-)	w[e] (mm s^{-1})	ϕ[f] (°)	θ[g] (°)	Red- χ^2 [h]
10 K	Fe(II)-O		12.4	1.84*	13.8*	-2.78	0.00*	0.30*	0*	90*	5.35
	Fe(II)-D1		21.1	1.32	0.0*	2.67	0.00*	0.30*	0*	0*	
	Fe(III)-S1		32.5	0.43	48.7	-0.01*	0.50*	0.49	0*	0*	
	Fe(III)-S2		11.9	0.41	42.7	0.03	0.00*	0.45	0*	0*	
	CF		22.1	0.50*	1.8	0.00*	0.00*	1.69	0*	0*	
≤ 5 K	Fe(II)-O	Fe(II) in green rust-like phase	32.0	1.67	12.7	-2.97	0.20*	0.40*	84*	95*	27.78
	Fe(II)-D1	Fe(II) sorbed onto Fe minerals and/or clays, or Fe(II) fraction of green rust before complete ordering of the octet	10.8	1.35	0.0*	2.71	0.00*	0.35*	0*	0*	
	Fe(III)-S1	Ferrihydrite and/or Fe(III) fraction of green rust	39.5	0.50	50.3	0.00	0.00*	0.47	0*	0*	
	Fe(III)-S2	Ferrihydrite and/or lepidocrocite	17.8	0.33	44.4	0.28	0.00*	0.50*	0*	0*	

[a]Center shift;
[b]Hyperfine field;
[c]Electric quadrupole interaction parameter
[d]Asymmetry parameter
[e]Half line width at half maximum
[f]Azimuthal angle between the electric field gradient (EFG) axis of symmetry with hyperfine field H
[g]Polar angle between the electric field gradient (EFG) axis of symmetry with hyperfine field
[h] Red-χ^2, goodness of fit;
* Indicates values that were fixed during the fitting process.

7.3 Additional soils incubation

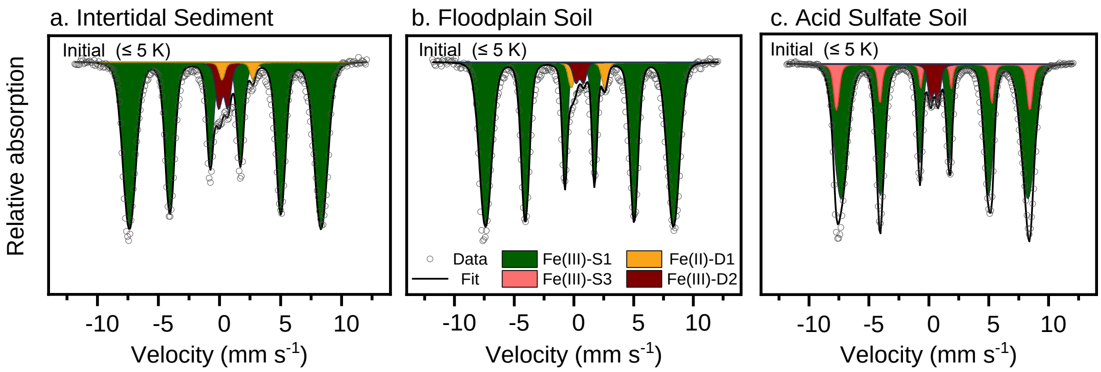

Figure S13. Fitted Mössbauer spectra of (a) Intertidal Sediment, (b) Floodplain Soil and (c) Acid Sulfate Soil spiked with ^{57}Fe-ferrihydrite before incubation. Spectra were collected at ≤ 5 K. Fitting parameters are detailed in **Tables S9.** Abbreviations: Fe(III)-D2 = Fe(III) doublet; Fe(II)-D1/2 = Fe(II) doublet; Fe(III)-S1/3 = Fe(III) sextet.

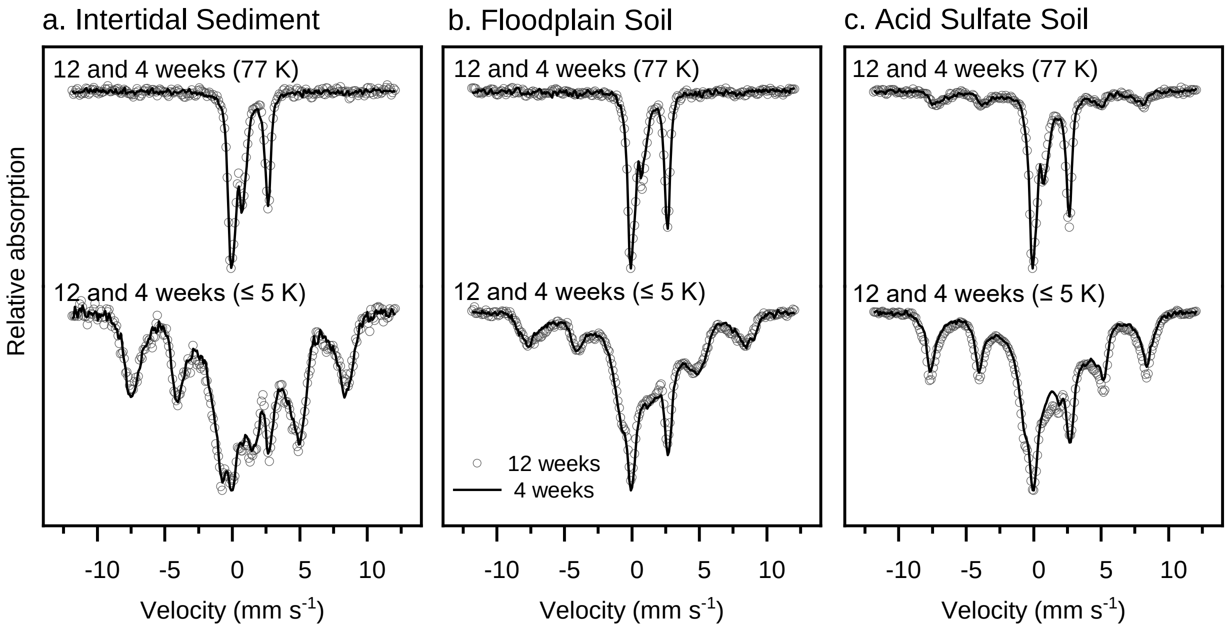

Figure S14. Mössbauer spectra of additional soils spiked with ^{57}Fe-ferrihydrite. Panels a, b, and c represent the different soils. The upper section plots data collected at 77 K, and the bottom section the data collected at 5 K. Data for 12-week samples is plotted as dots, while data for 4-week samples is plotted as a line. The data presented for 12 weeks is also shown in **Figure 4**.

Table S9. Hyperfine parameters obtained for fitting (xVBF model) of additional soils spiked with ^{57}Fe-ferrihydrite before and after 12-week incubation. Spectra collected at 77 K or ≤ 5 K (initial samples only).

Sample		Temp.	Phase	Phase interpretation (5 K only)	Spectral Area %	CS[a] (mm s^{-1})	QS or ε[b] (mm s^{-1})	H[c] (T)	σ[d] (mm s^{-1} or T)	Red- χ^2 [e]
Intertidal Sediment	Initial	77 K	Fe(III)-D1	-	94.2	0.47	1.17		0.96	0.71
			Fe(II)-D1	-	5.8	1.27	2.79		0.32	
		≤ 5 K	Fe(III)-D2	Fe(III) doublet from soil	6.4	0.46*	0.67		0.29*	6.25
			Fe(II)-D1	Fe(II) doublet from soil	2.2	1.16	3.06		0.30*	
			Fe(III)-S1	Added ferrihydrite phase	91.4	0.47	-0.00		48.67	
	12 W	77 K	Fe(III)-D1	-	52.2	0.47	0.78		0.47	0.58
			Fe(II)-D1	-	47.8	1.25	2.80		0.23	
Floodplain Soil	Initial	77 K	Fe(III)-D1	-	92.2	0.48	1.18		0.92	1.48
			Fe(II)-D1	-	7.9	1.21	2.97		0.38	
		≤ 5 K	Fe(III)-D2	Fe(III) doublet from soil	2.8	0.49*	0.67*		0.37*	5.29
			Fe(II)-D1	Fe(II) doublet from soil	3.6	1.19	2.79		0.40*	
			Fe(III)-S1	Added ferrihydrite phase	93.6	0.47	-0.01	48.8	2.83	
	12 W	77 K	Fe(III)-D1	-	44.6	0.51	0.82		0.57	0.85
			Fe(II)-D1	-	55.4	1.26	2.74		0.24	
Acid Sulfate Soil	Initial	77 K	Fe(III)-D1	-	87.6	0.46	1.26		1.01	6.27
			Fe(III)-S1	-	12.4	0.49	-0.16	47.2	3.01	
		≤ 5 K	Fe(III)-D2	Fe(III) doublet from soil	4.7	0.46	0.62		0.29*	38.64
			Fe(III)-S1	Added ferrihydrite phase	82.4	0.47	0.02	48.4	3.00	
			Fe(III)-S3	Sextet from soil (likely goethite)	12.9	0.48*	-0.12*	50.0	1.02	
	12 W	77 K	Fe(III)-D1	-	31.5	0.50	0.90		0.66	2.00
			Fe(II)-D1	-	46.9	1.26	2.72		0.30	
			Fe(III)-S3	-	10.6	0.51	-0.12	48.5	1.50	
			Fe(III)-S4	-	11.0	0.43	-0.03	43.7	3.00	

[a]Center shift;
[b]Quadrupole splitting (QS, for doublets) or quadrupole shift (ε, for sextets);
[c]Hyperfine field;
[d]σ, standard deviation of QS (doublets) or H (sextets);
[e]Red- χ^2, goodness of fit;

Abbreviations: Temp. = temperature; Fe(II)-D = Fe(II) doublet; Fe(III)-D = Fe(III) doublet; Fe(III)-S = Fe(III) sextet.
* Indicates values that were fixed during the fitting process.

Table S10. Hyperfine parameters obtained for fitting (Full Static Hamiltonian model) of additional soils with ^{57}Fe-ferrihydrite after 12-weeks incubation. Spectra collected at ≤ 5 K.

Sample		Temp.	Phase	Phase interpretation	Spectral Area %	CS[a] (mm s^{-1})	H[b] (T)	$e^2qQ/2$[c] (mm s^{-1})	η[d] (-)	w[e] (mm s^{-1})	ϕ[f] (°)	θ[g] (°)	Red- χ^2 [h]
Intertidal Sediment	12 W	≤ 5 K	Octet	Fe(II) in green rust-like phase	41.6	1.43	12.7	-3.08	0.2*	0.45*	60*	90*	2.88
			Fe(II)-D1	Fe(II) sorbed onto Fe minerals and/or clays, and/or Fe(II) fraction of green rust before complete ordering of the octet	11.5	1.24	0.0	2.97	0.0*	0.30*			
			Fe(III)-S1	Ferrihydrite and/or Fe(III) fraction of green rust	31.5	0.50	49.8	0.00*	0.0*	0.50			
			Fe(III)-S2	Ferrihydrite and/or lepidocrocite	12.5	0.50	44*	0.05*	0.0*	0.50*			
			Fe(III)-D2	Fe(III) surface-complexed and/or in phyllosilicates (possibly some pyrite) – likely component from the soil	3.9	0.50	0.0*	0.84	0.0*	0.20*			
Floodplain Soil	12 W	≤ 5 K	Octet	Fe(II) in green rust-like phase	41.1	1.37	10.6	-2.95	0.2*	0.50*	84*	95*	4.95
			Fe(II)-D1	Fe(II) sorbed onto Fe minerals and/or clays, and/or Fe(II) fraction of green rust before complete ordering of the octet	18.1	1.27	0.0*	2.76	0.0*	0.25*			
			Fe(III)-S1	Ferrihydrite and/or Fe(III) fraction of green rust	23.8	0.50*	50.1	0.00	0.0*	0.50*			
			Fe(III)-S2	Ferrihydrite and/or lepidocrocite	11.0	0.50*	43.7	0.10*	0.0*	0.50*			
			Fe(III)-D2	Fe(III) surface-complexed and/or in phyllosilicates (possibly some pyrite) – likely component from the soil	6.0	0.50*	0.0*	0.73	0.0*	0.25*			
Acid Sulfate Soil	12 W	≤ 5 K	Octet	Fe(II) in green rust-like phase	26.4	1.48*	11.8*	-2.99*	0.2*	0.50*	84*	95*	6.68
			Fe(II)-D1	Fe(II) sorbed onto Fe minerals and/or clays, and/or Fe(II) fraction of green rust before complete ordering of the octet	25.9	1.27	0.0*	2.78	0.0*	0.35*			
			Fe(III)-S1	Ferrihydrite and/or Fe(III) fraction of green rust	8.7	0.50*	46.3	0.00*	0.0*	0.47*			
			Fe(III)-S3	Mixture of goethite, nanogoethite and ferrihydrite	21.7	0.48*	50.0*	-0.12*	0.0*	0.30*			
			CF	Unknown	11.8	0.40*	21.0	-0.20	0.0*	0.60*			
			Fe(III)-D2	Fe(III) surface-complexed and/or in phyllosilicates (possibly some pyrite) - likely component from the soil	5.5	0.50*	0.0*	0.50*	0.0*	0.20*			

[a]Center shift;
[b]Hyperfine field;
[c]Electric quadrupole interaction parameter
[d]Asymmetry parameter
[e]Half line width at half maximum
[f]Azimuthal angle between the electric field gradient (EFG) axis of symmetry with hyperfine field H
[g]Polar angle between the electric field gradient (EFG) axis of symmetry with hyperfine field
[h] Red-χ^2, goodness of fit;
* Indicates values that were fixed during the fitting process.

8 PHOTOGRAPHIC RECORDS

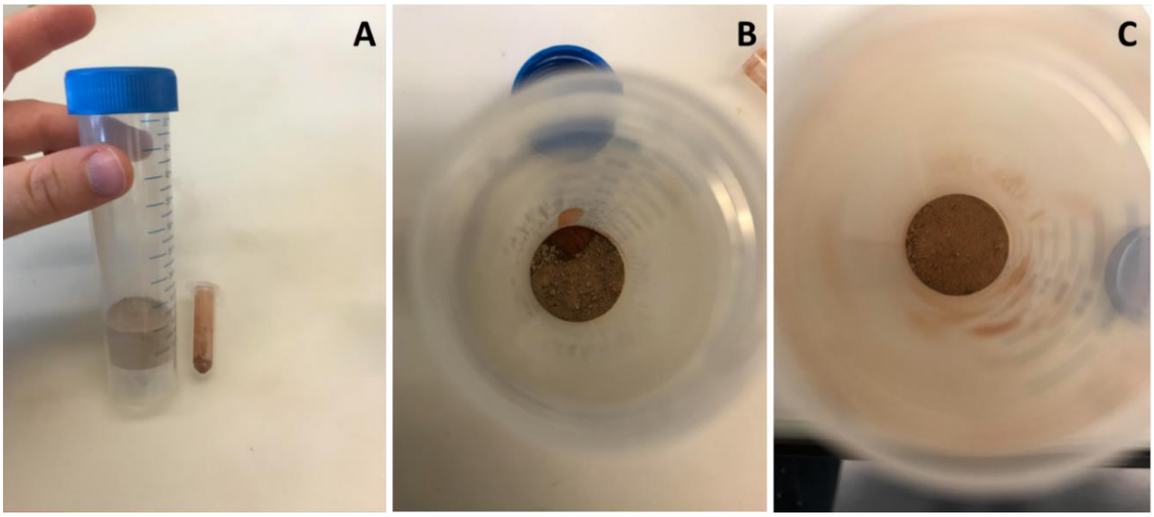

Figure S15. Paddy Soil and ^{57}Fe-ferrihydrite sample (a) separately, (b) directly after ^{57}Fe-ferrihydrite addition, and (c) after homogenization.

Photographic records of the experiment before (**Figure S15**) and after incubation (**Figure S16**) revealed that the flooding of the soil led to a clear aqueous phase with evidence of volume loss due to evaporation after 8 weeks. After the fourth week of incubation, a film was noticed at the water-air interface (**Figure S17**). At 16 weeks, after considerable water loss (estimated 1-2 mL), an 'oxidation ring' appeared in all tubes. Similar water loss and oxidation rings were observed in later samples of additional soils.

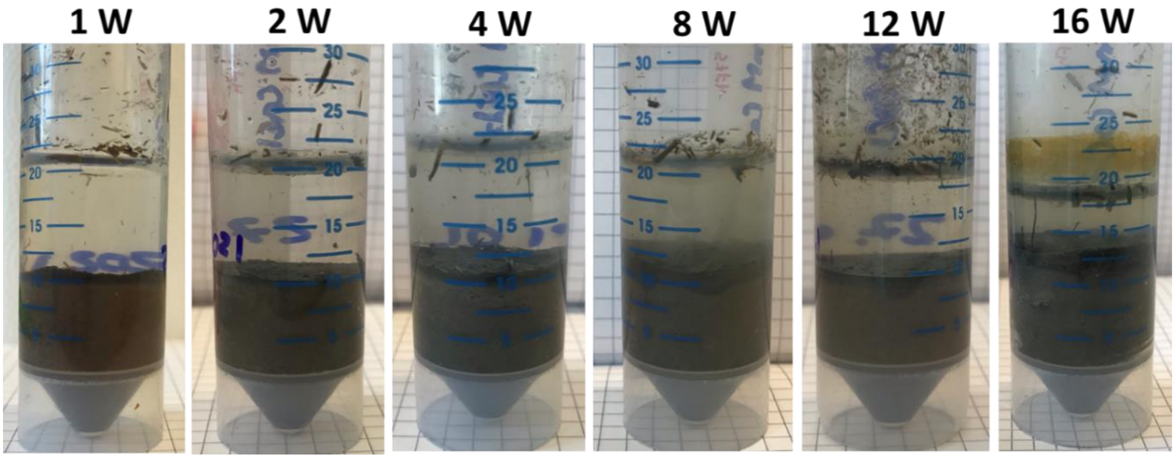

Figure S16. Microcosms of Paddy Soil incubated with ^{57}Fe-ferrihydrite at weeks 1-16.

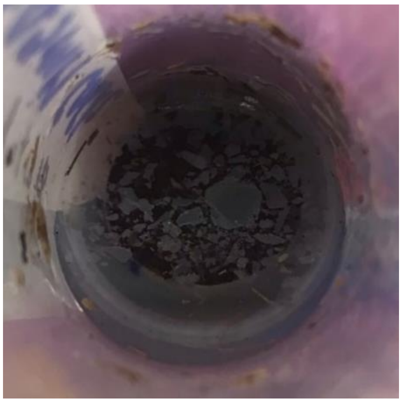

Figure S17. Microcosm of Paddy Soil incubated with ^{57}Fe-ferrihydrite at 4 weeks. This photo shows the inside of the tube under anoxic conditions, before any disturbances to the incubation other than removing the cover.

A comparison of photos from the experiment using 0.5 mM $CaCl_2$, parafilm, and non-agitation (**Figure S18a**) and microcosms incubated under varying conditions to test robustness at 4 weeks (**Figure S18b, c, and d**) revealed visual changes in the samples. Incubations with UPW water (**Figure S18b**) presented a grayish tone to the aqueous phase. Samples incubated with a closed cap instead of parafilm presented higher total volume, and therefore is evidence of the loss of aqueous phase in samples in the main experiment (e.g., 8 - 16 weeks) (**Figure S18c** compared to **Figure S16**). For agitated samples, suspended solids were observed in the aqueous phase (**Figure S18d**). For soils incubated without the addition of ferrihydrite, a yellowish color was observed in the aqueous phase (**Figure S18e**).

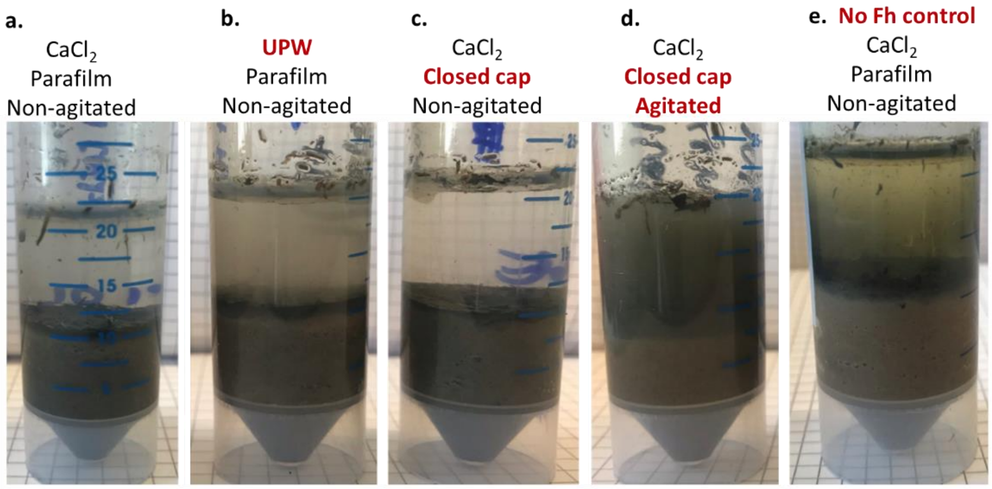

Figure S18. Microcosms of robustness test and no Fh control at 4 weeks.

The photographic records of incubations with additional soils (**Figure S19**) revealed an aqueous phase with more suspended solids compared to the Paddy Soil (**Figure S16**). Additionally, the Acid Sulfate Soil swelled upon the addition of the $CaCl_2$ solution, resulting in less aqueous phase available for sampling.

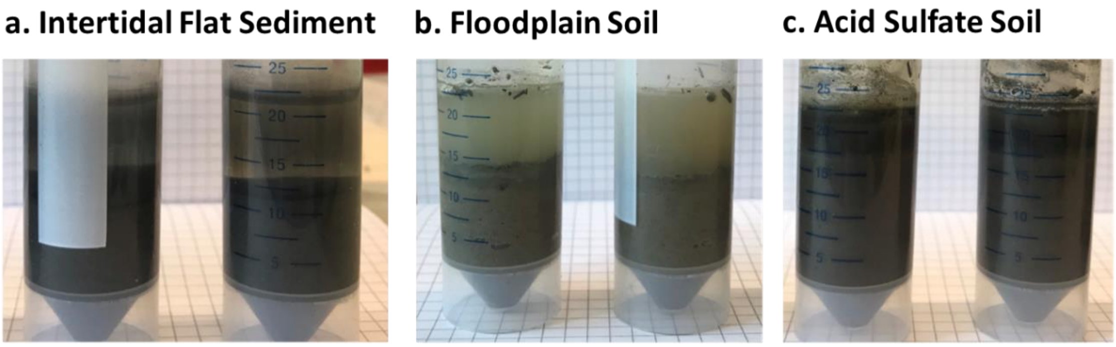

Figure S19. Microcosms of additional soils after four weeks of incubation. The photos display duplicates prepared with ^{57}Fe-ferrihydrite (left) and ^{NA}Fe-ferrihydrite (right).

9 REFERENCES

1. Tamura, H.; Goto, K.; Yotsuyanagi, T.; Nagayama, M., Spectrophotometric determination of iron(II) with 1,10-phenanthroline in the presence of large amounts of iron(III). *Talanta* **1974,** *21* (4), 314-318.
2. Schulz, K.; ThomasArrigo, L. K.; Kaegi, R.; Kretzschmar, R., Stabilization of ferrihydrite and lepidocrocite by silicate during Fe(II)-catalyzed mineral transformation: impact on particle morphology and silicate distribution. *Environ. Sci. Technol.* **2022,** *59* (9), 5929-5938.
3. Handler, R. M.; Beard, B. L.; Johnson, C. M.; Scherer, M. M., Atom exchange between aqueous Fe(II) and goethite: an Fe isotope tracer study. *Environ. Sci. Technol.* **2009,** *43* (4), 1102-1107.
4. Rancourt, D. G.; Ping, J. Y., Voigt-based methods for arbitrary-shape static hyperfine parameter distributions in Mössbauer spectroscopy. *Nucl. Instrum. Methods. Phys. Res. B* **1991,** *58* (1), 85-97.
5. Lagarec, K.; Rancourt, D. G., Extended Voigt-based analytic lineshape method for determining N-dimensional correlated hyperfine parameter distributions in Mössbauer spectroscopy. *Nucl. Instrum. Methods Phys. Res. B* **1997,** *129* (2), 266-280.
6. Blaes, N.; Fischer, H.; Gonser, U., Analytical expression for the Mössbauer line shape of ^{57}Fe in the presence of mixed hyperfine interactions. *Nucl. Instrum. Methods Phys. Res. B: Beam Interact. Mater. At.* **1985,** *9* (2), 201-208.
7. de Laeter, J. R.; Böhlke, J. K.; De Bièvre, P.; Hidaka, H.; Peiser, H. S.; Rosman, K. J. R.; Taylor, P. D. P., Atomic weights of the elements. Review 2000 (IUPAC Technical Report). **2003,** *75* (6), 683-800.
8. WRB, I. W. G., *World Reference Base for Soil Resources. International soil classification system for naming soils and creating legends for soil maps.* International Union of Soil Sciences Vienna, Austria, 2022; Vol. 4th edition.
9. Vandenberghe, R. E.; De Grave, E., Application of Mössbauer spectroscopy in earth sciences. In *Mössbauer Spectroscopy: Tutorial Book,* Yoshida, Y.; Langouche, G., Eds. Springer Berlin Heidelberg: Berlin, Heidelberg, 2013; pp 91-185.
10. Byrne, J. M.; Kappler, A., Mössbauer spectroscopy. In *Analytical geomicrobiology: A handbook of instrumental techniques,* Alessi, D. S.; Veeramani, H.; Kenney, J. P. L., Eds. Cambridge University Press: Cambridge, 2019; pp 314-338.
11. Dyar, M. D.; Schaefer, M. W.; Sklute, E. C.; Bishop, J. L., Mössbauer spectroscopy of phyllosilicates: effects of fitting models on recoil-free fractions and redox ratios. *Clay Miner.* **2008,** *43* (1), 3-33.
12. Thompson, A.; Chadwick, O. A.; Rancourt, D. G.; Chorover, J., Iron-oxide crystallinity increases during soil redox oscillations. *Geochim. Cosmochim. Acta* **2006,** *70* (7), 1710-1727.
13. Notini, L.; Byrne, J. M.; Tomaszewski, E. J.; Latta, D. E.; Zhou, Z.; Scherer, M. M.; Kappler, A., Mineral defects enhance bioavailability of goethite toward microbial Fe(III) reduction. *Environ. Sci. Technol.* **2019,** *53* (15), 8883-8891.
14. Murad, E.; Cashion, J., *Mössbauer spectroscopy of environmental materials and their industrial utilization*. Springer Science & Business Media: 2011.
15. Taylor, R. M., Influence of chloride on the formation of iron oxides from Fe(II) chloride. II. Effect of [Cl] on the formation of lepidocrocite and its crystallinity. *Clays Clay Miner.* **1984,** *32* (3), 175-180.
16. Thibeau, S.; Chiquet, P.; Mouronval, G.; Lescanne, M., Geochemical assessment of the injection of CO_2 into Rousse depleted gas reservoir. *Energy Procedia* **2009,** *1* (1), 3383-3390.

17. Zhou, Y.; Han, L. R.; He, H. W.; Sang, B.; Yu, D. L.; Feng, J. T.; Zhang, X., Effects of Agitation, Aeration and Temperature on Production of a Novel Glycoprotein GP-1 by *Streptomyces kanasenisi* ZX01 and Scale-Up Based on Volumetric Oxygen Transfer Coefficient. *Molecules* **2018,** *23* (1).
18. Dannenberg, S.; Wudler, J.; Conrad, R., Agitation of anoxic paddy soil slurries affects the performance of the methanogenic microbial community. *FEMS Microbiology Ecology* **1997,** *22* (3), 257-263.
19. Kukkadapu, R. K.; Zachara, J. M.; Fredrickson, J. K.; Kennedy, D. W., Biotransformation of two-line silica-ferrihydrite by a dissimilatory Fe(III)-reducing bacterium: formation of carbonate green rust in the presence of phosphate. *Geochim. Cosmochim. Acta* **2004,** *68* (13), 2799-2814.
20. Mackey, J.; Collins, R., The Mössbauer effect of iron in ion exchange resins. *J. Inorg. Nucl. Chem.* **1967,** *29* (3), 655-660.
21. Notini, L.; Latta, D. E.; Neumann, A.; Pearce, C. I.; Sassi, M.; N'Diaye, A. T.; Rosso, K. M.; Scherer, M. M., A closer look at Fe(II) passivation of goethite. *ACS Earth Space Chem.* **2019,** *3* (12), 2717-2725.
22. Génin, J.-M. R.; Abdelmoula, M.; Ruby, C.; Upadhyay, C., Speciation of iron; characterisation and structure of green rusts and FeII–III oxyhydroxycarbonate fougerite. *C. R. - Geosci.* **2006,** *338* (6-7), 402-419.
23. Thiel, J.; Byrne, J. M.; Kappler, A.; Schink, B.; Pester, M., Pyrite formation from FeS and H_2S is mediated through microbial redox activity. *Proc. Natl. Acad. Sci. U.S.A.* **2019,** *116* (14), 6897-6902.
